# Pressure-induced phosphorescence enhancement and piezochromism of a carbazole-based cyclic trinuclear Cu(i) complex†

**DOI:** 10.1039/d0sc07058k

**Published:** 2021-01-28

**Authors:** Mo Xie, Xiao-Ru Chen, Kun Wu, Zhou Lu, Kai Wang, Nan Li, Rong-Jia Wei, Shun-Ze Zhan, Guo-Hong Ning, Bo Zou, Dan Li

**Affiliations:** College of Chemistry and Materials Science, Guangdong Provincial Key Laboratory of Functional Supramolecular Coordination Materials and Applications, Jinan University Guangzhou Guangdong 510632 People's Republic of China guohongning@jnu.edu.cn danli@jnu.edu.cn; Department of Chemistry, Shantou University Shantou Guangdong 515063 People's Republic of China; State Key Laboratory of Superhard Materials, Jilin University Changchun 130012 People's Republic of China zoubo@jlu.edu.cn

## Abstract

Interest in piezochromic luminescence has increased in recent decades, even though it is mostly limited to pure organic compounds and fluorescence. In this work, a Cu_3_Pz_3_ (**Cu3**, Pz: pyrazolate) cyclic trinuclear complex (CTC) with two different crystalline polymorphs, namely **1a** and **1b**, was synthesized. The CTC consists of two functional moieties: carbazole (**Cz**) chromophore and **Cu3** units. In crystals of **1a**, discrete **Cz**–**Cu3**–**Cu3**–**Cz** stacking was found, showing abnormal pressure-induced phosphorescence enhancement (PIPE), which was 12 times stronger at 2.23 GPa compared to under ambient conditions. This novel observation is ascribed to cooperation between heavy-atom effects (*i.e.*, from Cu atoms) and metal–ligand charge-transfer promotion. The infinite π–π stacking of **Cz** motifs was observed in **1b** and it exhibited good piezochromism as the pressure increased. This work demonstrates a new concept in the design of piezochromic materials to achieve PIPE *via* combining organic chromophores and metal–organic phosphorescence emitters.

## Abstract 2

One molecule, two response mechanisms: a pair of newly-designed cyclic trinuclear Cu(i) complex crystalline polymorphs are engineered, which show excellent luminescent piezochromism and pressure-induced phosphorescence enhancement, respectively.

## Graphical abstract

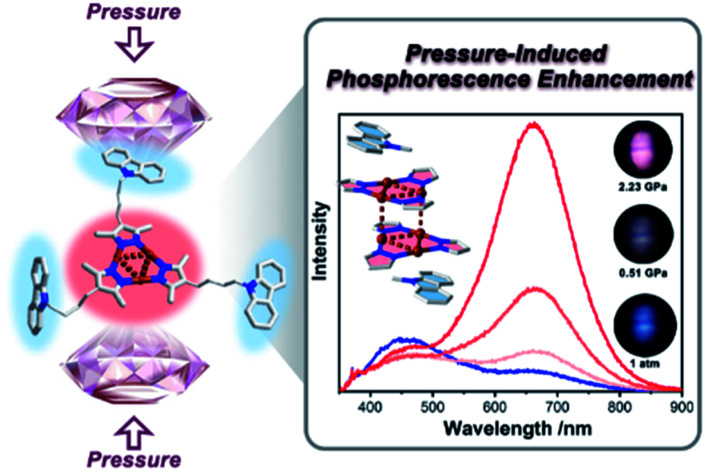

## Introduction

Piezochromic luminescent compounds are a class of stimuli-responsive materials^1,2^ which exhibit significant emission colour changes in response to grinding, pressing and stretching, and they show great potential in a wide range of applications, including pressure sensors, optical recording, and memory.^3–10^ Recent studies revealed that chromic mechanisms of such materials are related to pressure-controllable molecular switches,^11–13^ phase transition,^14,15^ conformational transformation,^16,17^ excited state transformation,^18^ and aggregation-induced emission (AIE) effect.^19,20^ To date, the molecular design and material selection for piezochromism are mostly focused on organic molecules.^21–25^ Because of the weak spin-orbital coupling and a long triplet-state lifetime, the pure organic molecules hardly show phosphorescence at room temperature (rt).^26,27^ Thus, most of piezochromic luminescent materials display a pressure-induced fluorescence change at rt (*e.g.*, quenched or enhanced intensity and bathochromic/hypsochromic shifts),^5,12,20^ whereas pressure-induced phosphorescence enhancement (PIPE) materials are highly challenging and still remain unexplored.

Cyclic trinuclear complexes (CTCs) with d^10^ metals are well known for their characteristic M_3_N_*x*_C_6−*x*_ (M = Au, Ag, or Cu; *x* = 0, 3, or 6) nine-membered ring and corresponding π-acidity/basicity, and metal–metal interactions (metallophilicity) which play essential roles in photoluminescence (PL) and other properties.^28,29^ In cooperation with other supramolecular interactions (non-covalent interactions),^30^ such stimuli-responsive luminescence has often been reported in the CTC family, including piezochromism,^31^ thermochromism,^32–34^ solvatochromism, and concentration luminochromism.^35^ In previous studies, phosphorescence of organic chromophores could be triggered at cryogenic temperatures or even at rt, after forming CTC/organic adducts or if directly serving as ligands.^28,36,37^ Such luminescence, termed “metal-sensitized phosphorescence”, is attributed to the enhanced intersystem crossing (ISC) rate from singlet excited states to triplet states mediated by an external heavy-atom effect (*e.g.*, Au, Ag, Cu and Hg).^38,39^ In this way, it is proposed that the external isotropic pressure can enforce the interactions between CTCs and organic chromophores, resulting in alteration of electronic structures of the emission centre and triggering the PIPE. As far as is known, investigations of the luminescence behaviour of such organic chromophore-CTC adducts under high pressure have rarely been reported.

**Scheme 1:**
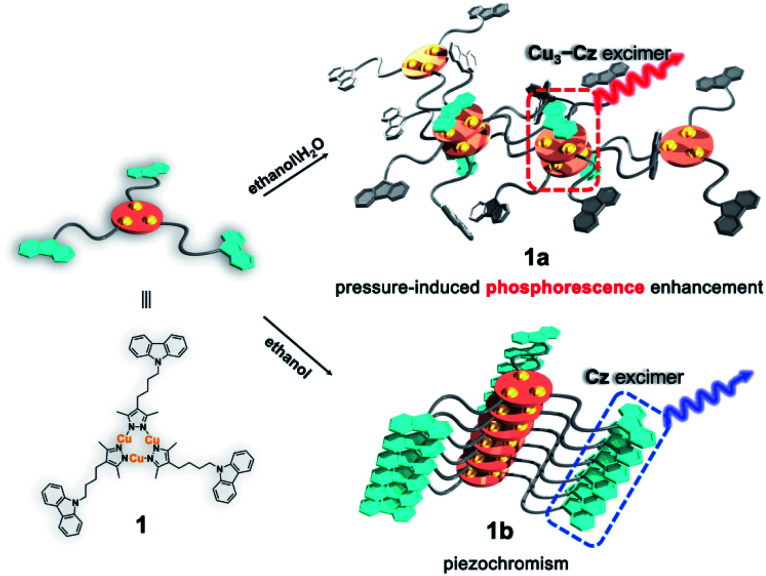
A schematic representation showing the molecular stacking of two crystalline polymorphs **1a** and **1b**, and the formation of the Cu_3_Pz_3_ CTC **Cz**–**Cu3**–**Cu3**–**Cz** excimer and carbazole (**Cz**) excimer with an increase in pressure.

In this work, a carbazole-based Cu(i) CTC (**1**) was designed by linking classic organic chromophore carbazole^40^ (**Cz**) and Cu_3_Pz_3_ CTCs (**Cu3** Pz: pyrazolate) with a non-conjugated flexible *n*-butyl chain. Two crystalline polymorphs, denoted as **1a** and **1b**, are obtained and exhibit two different stacking models leading to the formation of [**Cz**–**Cu3**–**Cu3**–**Cz**] excimers and **Cz** excimer/aggregator, respectively (Scheme 1). Therefore, metal-sensitized phosphorescence and the ligand-dominated luminescence are observed for **1a** and **1b**, respectively. Furthermore, **1a** shows an unprecedented PIPE phenomenon, which has never been reported in piezochromic materials, and **1b** exhibits conventional carbazole characteristic piezochromic luminescence with a good linear relationship of intensity *versus* external pressure.

## Results and discussion

**Fig. 1:**
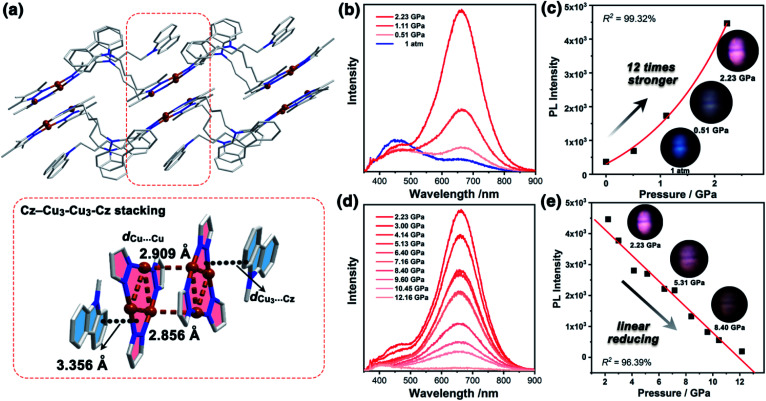
(a) The crystal packing of **1a** at 100 K, showing discrete **Cz**–**Cu3**–**Cu3**–**Cz** stacking (Cu: brown, C: grey, and N: blue, H atoms are omitted for clarity). Emission spectra of **1a** at a range of external pressures from (b) 1 atm–2.23 GPa and (d) 2.23–12.16 GPa at an excitation wavelength of 355 nm. The curve fitting [function: *y* = *A*_1_ × exp(−*x*/*t*_1_) + *y*_0_, *R*^2^ = 99.32%] and linear fitting [function: *y* = *A*_0_ + *kx*, *R*^2^ = 96.39%] of pressure and PL intensity for **1a** in pressure ranges of (c) 1 atm–2.23 GPa and (e) 2.23–12.16 GPa (insets of (c) and (e): PL photographs at representative pressure points).

**Fig. 2:**
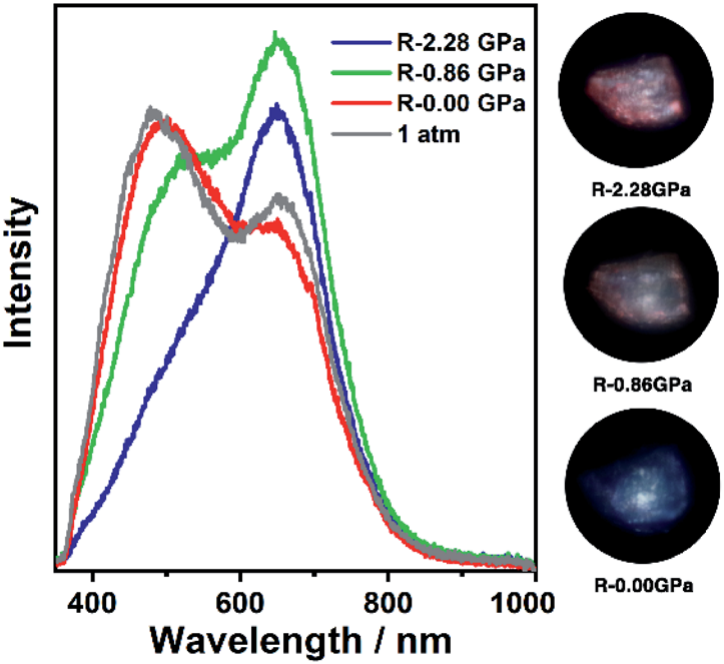
The PL spectra and photographs of **1a** in a pressure relief process from 2.28 GPa to 0.00 GPa. The gray line (marked as 1 atm) is the PL spectrum before compression.

The yellow block-shaped crystals of **1a**, or colourless fibrous crystals of **1b** were obtained by mixing 9-(4-(3,5-dimethyl-1*H*-pyrazol-4-yl)butyl)-carbazole ligand (**HL**) and Cu_2_O in anhydrous ethanol or ethanol/water under solvothermal conditions (see ESI† for further details). The freshly synthesized single-crystal samples of **1a** and **1b** were characterized by powder X-ray diffraction (PXRD) and infrared (IR) spectroscopy, confirming their phase purity (Fig. S7 and S8, ESI†). The single X-ray crystallographic analysis of **1a** and **1b** revealed that both featured the nine-membered Cu_3_N_6_ CTC composed of **Cu3** units and **Cz** motifs (Fig. 1a and 2a).

Unlike the conventional sandwich-like ternary adducts (*i.e.*, CTC–organic aromatic–CTC),^41,42^ unprecedented discrete adducts with a **Cz**–**Cu3**–**Cu3**–**Cz** stacking model were observed in **1a** (Fig. 1a). Specifically, two adjacent **Cu3** units adopted a dimer-of-trimer configuration with short intertrimer *d*_Cu⋯Cu_ of 2.854 and 2.909 Å, implying that there were strong Cu⋯Cu interactions between the **Cu3** units. Moreover, one set of **Cz** groups adducted with the **Cu3**–**Cu3** dimer and the intermolecular plane-to-plane distance between the **Cu3** unit and the **Cz** group (*d*_Cu_3_⋯Cz_) was 3.360 Å, which indicated weak π-acid⋯base interactions (Fig. 1a). It is worth noting that the other **Cz** groups were randomly arranged and no noticeable interactions between **Cu3** or **Cz** units were observed (Fig. S10 and S11, ESI†).

**1a** exhibits a UV-vis absorption profile similar to that of the proligand **HL** in either CH_2_Cl_2_ solution or solid state (Fig. S14 and S15, ESI†). The absorption bands of **1a** at about 290 and 340 nm can be attributed to ligand-centred (LC) transition. The variable-excitation wavelength PL spectra of **1a** at rt were remarkably different from those of the proligand (Fig. S16 and S17, ESI†). As shown in Fig. S18 (ESI†), **1a** has low-energy (LE) dominated dual emission bands at about 400 nm (high-energy, HE band) and 680 nm (LE band) with a large LE/HE intensity ratio of 100 (*λ*_ex_ = 280 nm). The HE band was consistent with the solid emission of the proligand in both emission energy and band profile, which can be assigned as a LC fluorescence. The LE band intensity was significantly larger compared with that of the proligand, and it remained at a certain strength even under LE excitation (*i.e.*, *λ*_ex_ = 360 nm), indicating that the LE band was not limited to the emission of the proligand. Upon increasing the excitation energy, the emission colour changed from blue-white to red (Fig. S18b, ESI†) in the CIE-1931 chromaticity diagram.

Upon cooling to 200 K or a lower temperature, **1a** showed a new broad emission band at 560 nm, whose emission energy was temperature-independent (Fig. S20a, ESI†). To better understand the steady state PL, density functional theory (DFT) and time-dependent density functional theory (TDDFT) calculations were conducted and the calculations based on singlet monomeric **1a** geometry revealed that the low-lying singlet excited states were mainly localized on the **Cz** moieties, until the metal-to-ligand charge transfer (MLCT) character became dominant in the S_14_ state (Table S4, ESI†). These results suggested that **1a** will produce ^1^LC fluorescence upon LE excitation to the S_1_–S_13_ states. By exciting **1a** to the S_14_ or higher energy singlet states by increasing excitation energy, the ISC process might be generated by metal copper participation. The **Cz**–**Cu3**–**Cu3**–**Cz** stacking model of **1a** is divided into two theoretical models for clarity, denoted as dimer (a) or (b), which contained **Cu3**–**Cu3** dimers or **Cu3**–**Cz** adducts, respectively. The lowest single–triplet excited states of both monomer and dimer (a) were localized on the **Cz** moieties (Table S5, ESI†), which can be assigned as metal-sensitized ligand centred (MSLC) phosphorescence. The charge transfer (^3^LC/^3^MLCT) excited state based on the **Cu3**–**Cz** interaction contributed a little (9%) to the triplet emission of dimer (b), suggesting that the copper metal directly participated in the triplet electronic transition rather than just sensitizing the LC emission. Therefore, the LE dual emissions of **1a** can be assigned as the ^3^LC of the monomer (*λ*_em_ = 560 nm) and the domination of the MSLC mixed with the ^3^MLCT assisted phosphorescence of the **Cz**–**Cu3**–**Cu3**–**Cz** excimer (*λ*_em_ = 680 nm). Taking advantage of the **Cu3**–**Cz** interactions, the heavy atom effects of copper atoms promoted the ISC rate and the generation of low-energy phosphorescence, leading to MSLC phosphorescence dominated emission of **1a**. The proposed complete photophysical processes are summarized in Fig. S23 (ESI†).

It is known that pure carbazole possesses excellent piezoluminochromism.^40^ The unique mixed MSLC/MLCT phosphorescence of **Cz**-based CTC **1a** promoted further study of the PL under high pressure. The isotropic hydrostatic pressure was directly applied *via* a diamond anvil cell (DAC)^43^ on crystal samples of **1a** (see ESI† for experimental details). At ambient conditions, **1a** exhibited LC dominated blue emission, under LE excitation (355 nm). As the external pressure increased, **1a** presented constant fluorescence reduction and phosphorescence enhancement. The intensity of the LE phosphorescence showed exponential growth and was greatly increased: 12 times stronger at 2.23 GPa than that at 1 atm (10^−4^ GPa), whereas the HE emission intensity was slightly weakened, resulting in a remarkable colour change from weak blue to bright pink (Fig. 1b and c). Such PIPE is rare in the piezochromic luminescent materials. When the pressure was greater than 2.23 GPa, the emission intensity decreased linearly (Fig. 1d, e and S24, ESI†). The reduced LE emission and enhanced HE emission recorded during a pressure relief process from 2.28 GPa to 0 GPa (Fig. 2) indicated that the PIPE phenomenon of **1a** was reversible. To further explain these results, TDDFT calculations were performed based on the T_1_ geometry of **1a** at 1 atm and 2.5 GPa (Fig. S28–S30, ESI†). Under ambient conditions, the T_1_ state was mainly composed of ^3^LC transition of **Cz** moieties (79%) and LLCT (ligand-to-ligand charge transfer) transition of **Cu3**–**Cz** (9%). Pressurizing **1a** to 2.5 GPa, the ^3^MLCT/^3^LLCT transition became dominant in the T_1_ state (65%) while the percentage of ^3^LC transition of **Cz** moieties was reduced to 14%. In addition, when the pressure increased from 1 atm to 2.5 GPa, the Cu contribution in the MLCT increased from 6.46% to 11.75%. These computational results indicated the external pressure strength of the **Cu3**–**Cz** interactions, resulting in the increase of the ^3^MLCT/^3^LLCT contribution in the excited state. Combining the crystal structure analysis and the computational results, it can be postulated that the weak LE emission band is attributed to MSLC phosphorescence due to the rather weak **Cu3**–**Cz** interactions and the negligible Cu contribution to the MLCT state at ambient conditions. When the pressure was increased to 2.23 GPa, the intensity of the LE phosphorescence was greatly enhanced, which could reasonably be attributed to a cooperative effect between heavy-atom effects and MLCT promotion because of the shortening of *d*_Cu3⋯Cz_ as well as the significant increase of **Cu3**–**Cz** interactions and Cu contribution in the MLCT (Fig. 4a).

**Fig. 3:**
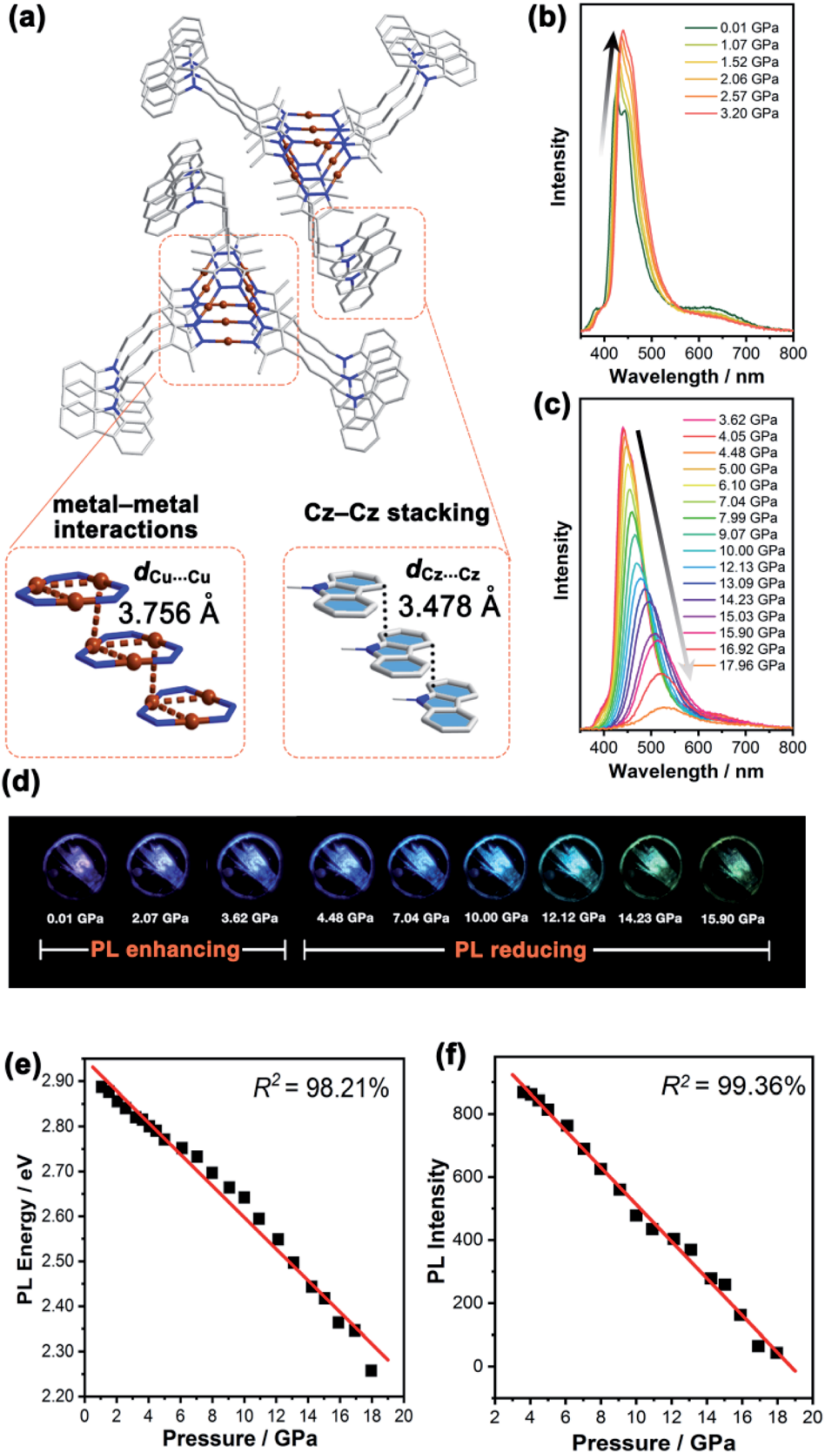
(a) The crystal packing of **1b** at 298 K, showing the infinite chain stacking model of **Cz** and **Cu3**. The emission spectra of **1b** at a range of external pressures from (b) 0.01–3.20 GPa and (c) 3.62–17.96 GPa at an excitation wavelength of 355 nm. (d) PL photographs of the **1b** crystal under compression up to 15.90 GPa. Linear fitting of the external pressure and (e) PL energy and (f) PL intensity of **1b**.

In contrast, the **Cu3** units in **1b** packed as a stair-step infinite chain with one pair of weak intertrimer Cu⋯Cu interactions with a relatively long intermolecular Cu⋯Cu distance (*d*_Cu⋯Cu_) of 3.756(3) Å at 298 K (Fig. 3a). In addition, the **Cz** motifs adopted a *J*-type aggregation along the *a*-axis, whereby the two adjacent **Cz** groups tightly stacked through π–π interactions with an intermolecular **Cz**⋯**Cz** plane-to-plane distance (*d*_Cz⋯Cz_) of about 3.480 Å (Fig. 3a). **1b** presented different intensity ratios of higher and lower absorption bands in UV-vis absorption compared with that of **1a**, which explained the different crystal colours of **1a** and **1b** (Fig. S12 and S13, ESI†). In sharp contrast, **1b** shows HE dominated dual emission with a much smaller LE/HE intensity ratio of 1.25 (*λ*_ex_ = 280 nm) than that of **1a** (Fig. S19a, ESI†). The HE structured bands in the range of 350–500 nm were similar to those for the proligand (Fig. S16 and S17, ESI†) which can be attributed to the LC emission upon LE excitation (*i.e.*, *λ*_ex_ = 360 nm). The excitation wavelength-dependent emission colour changes were also found for **1b** (Fig. S19b, ESI†). The DFT and TDDFT calculations revealed that the low-lying singlet excited states were **Cz** localized, confirming the ^1^LC feature of the HE emission of **1b**. Both the lowest triplet states of monomeric and dimeric **1b** were a ^3^LC feature (Table S6, ESI†), therefore the LE emission band of **1b** can be attributed to the MSLC phosphorescence which experiences the ISC from the ^1^MLCT state to the ^3^MLCT state and the internal conversion (IC) of the triplet state. Such a proposed photophysical mechanism has been commonly observed in other CTC systems.^44^ Because of the well-ordered *J*-type aggregation of the **Cz** motifs, **1b** exhibited aggregate/excimer emission with a lower energy in the solid state, which further supported the lower energy broad emission of **1b** compared to that of the proligand. Combining the experimental and computational studies, **1a** showed phosphorescence dominated emission, but **1b** featured similar emission behaviours to the proligand, revealing that the stacking mode of the organic chromophore in the crystals would be an essential factor for determining the PL properties of the metal–organic complex.

**Fig. 4:**
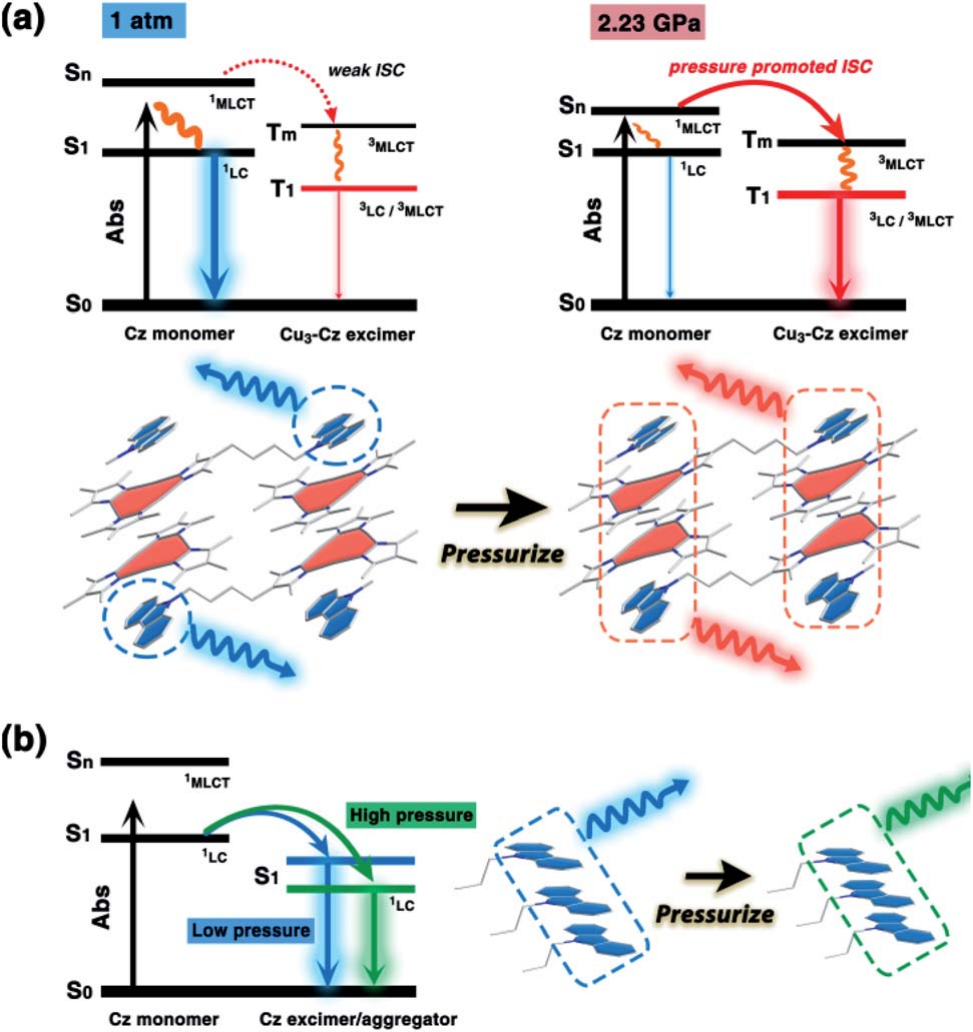
A schematic diagram of the (a) pressure-induced phosphorescence enhancement of **1a**, and (b) the piezochromism of **1b**. The dominant luminescent components are highlighted.

Upon external pressure, the PL of **1b** showed a continuous red shift, accompanied by two-step changes including emission enhancement (0.01–3.62 GPa) and emission reduction (3.62–17.96 GPa) under 355 nm excitation (Fig. 3b–d). These observations were similar to the piezoluminochromism of pure **Cz** (ref. 40) and agreed well with the previously mentioned suggestion for the **Cz**-centred characteristic luminescence of **1b**. Interestingly, both PL energy and intensity (or the integrated area in Fig. S25, ESI†) showed a linear relationship *versus* the external pressure as shown in Fig. 3e and f, respectively. The emission spectra and photographs taken during the decompression process indicate that the piezoluminochromic phenomena of **1b** (Fig. S26, ESI†) was reversible. The pressure-dependent structural simulations and frontier molecular orbital (FMO) analysis (Fig. S30–S34, ESI†) of **1b** revealed that the *d*_Cz⋯Cz_ was the only structural parameter to change consistently with the cell volume change. It is a key factor that influences the overlap of molecular orbitals participating in the PL transition, leading to a continuous bathochromic shift in the PL energy. As the **Cz** groups were well separated from the **Cu3** moieties with non-conjugated *n*-butyl chains and well packed to form an infinite and column-like **Cz** aggregator *via* π–π interactions in the crystals, **1b** mainly exhibited the **Cz**/**Cz** excimer-based luminescence and the contribution of MLCT to the PL were negligible (Fig. 4b), and which are not favoured for d^10^-metal CTCs.^39^

## Conclusions

In summary, a new piezochromic material, carbazole-based Cu(i) CTCs, consisting of a carbazole chromophore and Cu_3_Pz_3_ functional moieties linked by a non-conjugated alkyl chain, was designed. Significant stacking conformations were observed in the two crystalline polymorphs of **1a** and **1b**: discrete **Cu3**–**Cz** adducts *via* π-acid⋯base interactions were found in **1a**, whereas the infinite π–π stacking of **Cz** groups and a stair-step infinite chain of **Cu3** motifs were obtained in **1b**. Like pure carbazole molecules, upon increasing the external pressure, the emission maximum of **1b** gradually red-shifted and the intensity of the fluorescence band slightly increased before gradually decreasing. However, the unprecedented phenomenon of the pressure-induced phosphorescence enhancement (PIPE) of **1a** was discovered, which was a rare case in the field of piezochromic luminescence. Theoretical and experimental investigations illustrated that the increase of the LE phosphorescence band intensity originated from the **Cu3**–**Cz** excimer in **1a**. External heavy atom effects induced MSLC phosphorescence and then promoted MLCT events from the **Cu3** unit to the **Cz** groups cooperatively contributed to the enhancement of the LE phosphorescence band from 1 atm to 2.5 GPa. These studies demonstrated a novel strategy for designing a new class of piezochromic materials with PIPE properties *via* the combination of external heavy atom effects and MLCT promotion. The excellent responses to pressure of **1a** and **1b** make them promising materials for use as pressure sensors or in pressure detection.

## Experimental section

### Materials

All starting materials were purchased from commercial sources and used as received without further purification. Detailed characterization methods are included in the ESI.†

#### Synthesis of the 9-(4-(3,5-dimethyl-1*H*-pyrazol-4-yl)butyl)-carbazole ligand (**HL**)

Carbazole (10.32 g) was dissolved in 100 mL of toluene, and the solution was added to 1,4-dibromobutane (118.2 g, 547.4 mmol) and tetrabutylammonium bromide (TBAB, 2 g) in a 500 mL round bottom flask, and then 100 mL of 50% sodium hydroxide solution was added into the mixed solution, and the stirring was continued at 45 °C for 24 h. The mixed solution was extracted with dichloromethane, the extraction solvent was then steamed and placed under a fume hood and allowed to volatilize for a week. After evaporation to dryness, the blue solid obtained was separated and purified by column chromatography to obtain a large amount of a white solid, which was intermediate **HL-1**: 9-(4-bromobutyl)-9*H*-carbazole with a yield of about 86%. ^1^H-NMR (400 MHz, CD_2_Cl_2_) *δ* 8.16 (d, *J* = 7.8 Hz, 1H), 7.50 (m, 2H), 7.28 (dd, *J* = 7.9, 6.8 Hz, 1H), 4.39 (t, *J* = 7.2 Hz, 1H), 3.44 (t, *J* = 6.5 Hz, 1H), 2.07 (m, 1H), 1.95 (m, 1H) ppm. ^13^C-NMR (400 MHz, CD_2_Cl_2_) *δ* 140.3, 125.7, 122.8, 120.3, 118.9, 108.7, 42.2, 33.4, 30.4, 27.6 ppm. Elemental analysis for C_16_H_16_BrN, calcd (%): C 63.59, H 5.34, N 4.62; found (%): C 64.07, H 5.63, N 4.58.

The eluent selected was dichloromethane : petroleum ether (1 : 8). Next K_2_CO_3_ (80 mol, 11.04 g), acetylacetone (60 mmol, 6 mL), and a small amount of 18-crown-6 was added to a three-necked round bottom flask containing 100 mL of acetone. Then 1-bromobutyl 4-oxazole (20 mmol, 6.038 g) was dissolved in 60 mL of acetone and placed in a dropping funnel and attached to a three-necked flask. After installing the device, nitrogen gas was added and the mixture was stirred at rt for 30 min. After 30 min, the temperature was raised to 50 °C, and the 1-bromobutyl 4-oxazole solution in the dropping funnel was gradually added dropwise to the three-necked flask, and the mixture was heated to reflux, and the nitrogen was left flowing for 17 h. After the reaction was completed, distilled water was added, and the mixture was extracted with a dichloromethane solution, and then steamed, and purified by column chromatography. The eluent selected was dichloromethane : petroleum ether (1 : 10) and colorless needle-like crystals were obtained, which was the intermediate **HL-2**: 3-(4-(9*H*-carbazol-9-yl)butyl)pentane-2,4-dione with a yield of about 51%. ^1^H-NMR (400 MHz, CD_2_Cl_2_) *δ* 8.13 (d, *J* = 7.8 Hz, 1H), 7.48 (m, 2H), 7.26 (dd, *J* = 7.4, 6.9 Hz, 1H), 4.35 (t, *J* = 7.2 Hz, 1H), 3.58 (t, *J* = 7.1 Hz, 1H), 2.12 (m, 3H), 1.92 (m, 1H), 1.84 (m, 1H), 1.35 (m, 1H) ppm. ^13^C-NMR (400 MHz, CD_2_Cl_2_) *δ* 204.0, 140.4, 125.6, 122.7, 120.2, 118.8, 108.7, 68.1, 42.7, 29.2, 28.8, 27.8, 25.3 ppm. Elemental analysis for C_21_H_23_NO_2_, calcd (%): C 78.47, H 7.21, N 4.36; found (%): C 78.67, H 7.40, N 4.37.

The diketone intermediate was added to a flask containing hydrazine hydrate (80%, 14 mL), ethanol (100 mL), and kept at 70 °C for 15 h, and the ethanol evaporated to give a yellow oil. After freezing in a refrigerator for one week, the yellow oil was taken out, and a white solid was observed. After washing with a diethyl ether solution, it was filtered and dried to give a product **HL** [9-(4-(3,5-dimethyl-1*H*-pyrazol-4-yl)butyl)-9*H*-carbazole] with a yield of about 39%. ^1^H-NMR (400 MHz, CD_2_Cl_2_) *δ* 8.13 (d, *J* = 7.8 Hz, 1H), 7.50 (m, 2H), 7.25 (dd, *J* = 7.9, 6.7, 1H), 4.35 (t, *J* = 7.2 Hz, 1H), 2.38 (t, *J* = 7.6 Hz, 1H), 2.13 (s, 3H), 1.90 (m, 1H), 1.56 (m, 1H) ppm. ^13^C-NMR (400 MHz, CD_2_Cl_2_) *δ* 141.8, 140.4, 125.6, 122.7, 120.2, 118.7, 115.0, 108.7, 43.0, 28.7, 28.2, 22.8, 10.6 ppm. IR spectrum (KBr, cm^−1^): 3203 (w), 3147 (w), 3089 (w), 3042 (w), 3007 (w), 2928 (m), 2856 (w), 1626 (m), 1592 (m), 1482 (m), 1451 (m), 1413 (m), 1383 (w), 1325 (m), 1298 (w), 1238 (m), 1206 (m), 1177 (w), 1153 (m), 1104 (w), 1066 (w), 1020 (w), 1000 (w), 924 (w), 901 (w), 850 (w), 771 (w), 749 (s), 721 (s), 628 (w), 615 (w), 556 (w), 527 (w), 445 (w), 421 (m). Elemental analysis for C_21_H_23_N_3_, calcd (%): C 79.46, H 7.30, N 13.24; found (%): C 79.61, H 7.41, N 13.41. Detailed characterization of **HL** and the intermediates are included in the ESI.†

### Synthesis of **1a**

A solvo thermal method^45^ was used to synthesize the target complex **1a** and **1b**. The Cu_2_O (4.32 mg, 0.03 mmol), **HL** (9.51 mg, 0.03 mmol), 1.5 mL of ethanol and 1.5 mL of water were added to a hard glass tube with an inner diameter of 8 mm, and the tube was sealed and heated to 140 °C for 72 h. After cooling the tube and its contents to rt at a rate of 3 °C per h, the contents were filtered and washed with ethanol to give a light yellow block crystal with a yield of about 52%. Elemental analysis for C_63_H_66_N_9_Cu_3_, calcd (%): C 66.15, H 6.06, N 10.74; found (%): C 66.38, H 5.84, N 11.06.

### Synthesis of **1b**

The Cu_2_O (4.22 mg, 0.03 mmol), **HL** (9.51 mg, 0.03 mmol) and 3 mL of ethanol were added to a hard glass tube with an inner diameter of 8 mm, and the tube was sealed and heated to 140 °C for 72 h. After cooling the tube and its contents to rt at a rate of 3 °C per h, the contents were then filtered and washed with ethanol to give a colorless filamentous crystal with a yield of about 70%. Elemental analysis for C_63_H_66_N_9_Cu_3_, calcd (%): C 66.38, H 5.84, N 11.06; found (%): C 66.12, H 5.65, N 10.96.

### Crystallographic studies

Single-crystal X-ray diffraction (SCXRD) data for **1a** and **1b** were collected by an Cryostream system (Oxford Cryosystems) on a XtaLAB PRO MM007 DW diffractometer system equipped with a RA-Micro7HF-MR-DW(Cu/Mo) X-ray generator and a Pilatus3R-200K-A detector (Rigaku, Japan), Cu Kα, *λ* = 1.54178 Å. The numerical absorption corrections were applied using the ABSCOR program. The data set temperatures were 296 K and 100 K for **1a** and 298 K for **1b**. For **1a**, the structures were solved using direct methods, which yielded the positions of all the non-hydrogen atoms. These were first refined isotropically and then anisotropically. All the hydrogen atoms of the ligands were placed in calculated positions with fixed isotropic thermal parameters and included in the structure factor calculations in the final stage of the full-matrix least-squares refinement.

For **1b**, the low quality of the crystal structure of the sample was due to poor data and a disorder problem of the flexible *n*-butyl chain and the carbazole ring, respectively. The cell parameters and atomic position of the CTC rings and heavy atom were generated from the SCXRD data by the Patterson method.

All the calculations were performed using the system of computer programs.^46,47^ The crystal data and structure refinement parameters are summarized in Table S1 (ESI†). Selected bond lengths and angles are given in Table S2 (ESI†).

## Conflicts of interest

There are no conflicts to declare.

## Supplementary Material

SC-012-D0SC07058K-s001

SC-012-D0SC07058K-s002
