# Supplementary material for: Pressure-induced phosphorescence enhancement and piezochromism of a carbazole-based cyclic trinuclear Cu(i) complex
Source: Chem Sci. 2021 Jan 28;12(12):4425–31. doi: 10.1039/d0sc07058k (PMC8179561; doi:10.1039/d0sc07058k)
Supplement: SC-012-D0SC07058K-s001 [file SC-012-D0SC07058K-s001.pdf]

Electronic Supplementary Material (ESI) for Chemical Science.
This journal is © The Royal Society of Chemistry 2021

Electronic Supplementary Information

Pressure-Induced Phosphorescence Enhancement and Piezochromism of a Carbazole-based Cyclic Trinuclear Cu(I) Complex

Mo Xie,[a] Xiao-Ru Chen,[b] Kun Wu,[a] Zhou Lu,[§a] Kai Wang,[c] Nan Li,[c] Rong-Jia Wei,[a] Shun-Ze Zhan,[b] Guo-Hong Ning,*[a] Bo Zou,*[c] and Dan Li*[a]

[a.] *College of Chemistry and Materials Science, Guangdong Provincial Key Laboratory of Functional Supramolecular Coordination Materials and Applications, Jinan University, Guangzhou, Guangdong 510632, People's Republic of China E-mail: guohongning@jnu.edu.cn, danli@jnu.edu.cn.*

[b] *Department of Chemistry, Shantou University, Shantou, Guangdong 515063, People's Republic of China.*

[c] *State Key Laboratory of Superhard Materials, Jilin University, Changchun 130012, People's Republic of China E-mail: zoubo@jlu.edu.cn*

§ Present address: Department of Chemistry, University of North Texas, Denton, Texas 76203, USA.

*Corresponding Author:

G.-H. Ning guohongning@jnu.edu.cn; B. Zou zoubo@jlu.edu.cn; D. Li danli@jnu.edu.cn.

Experimental Section

Materials and Physical Measurements

All starting materials were purchased from commercial sources and used as received without further purification.

The D8 Advance X-ray diffractometer (Cu target) was used to characterize the crystal phase purity of the complex. An Elementar Vario EL III CHNS elemental analyzer was used to analyze the C, H, and N elements of the complex. The Nicolet Avatar 360 infrared spectrometer and KBr tablet were used to perform fourier-transform (FT-IR) spectra of the complexes. The range of 4000-400cm^{-1} was to scan the complexes by infrared characterization. Solution UV-vis absorption spectra were recorded on an Agilent UV-vis spectrometer 8453; solid-state UV-Vis absorption spectra were recorded on a Bio-Logic MOS-450 multifunctional circular dichroism spectrometer. The thermogravimetric analysis (TGA) of the complex was characterized on a TA Instruments Q50. Temperature rise rate at N_2 flow rate 40-60mL/min is 10℃/min, test temperature range is room temperature -800.

Steady-state photoluminescence spectra (PL) for all samples were tested on a PTI Quanta Master Model QM/TM fluorescence spectrometer (Birmingham, NJ, USA), and the detector was calibrated using a calibration file in the FelixGX software. The temperature change spectrum test uses the Janis Research Model VPF-100 temperature change system with liquid nitrogen Dewar, the temperature range is 77-450 K, and the interval is 25 K. In the test of the temperature-variable steady-state spectrum, ensure that the instrument parameters are consistent. The lifetime was tested by Edinburgh FLS-920 fluorescence spectrometer. The absolute photoluminescence quantum yields were measured by employing Hamamatsu C11347-01 absolute PL quantum yield spectrometer under both room temperature and liquid nitrogen.

General Synthesis and Characterization

***H*L：** 10.32 g carbazole was dissolved in 100 mL of toluene, and the solution was added to 1,4-dibromobutane (118.2 g, 547.4 mmol) and tetrabutylammonium bromide (TBAB, 2 g) in a 500 mL round bottom flask, then add 100 mL of 50% sodium hydroxide solution into the mixed solution, keep stirring at 45 °C for 24 h. The mixed solution was extracted with dichloromethane, steam the extracted solvent and place in a fume hood and volatile for a week. After evaporation to dryness, the obtained blue solid was separated and purified by column chromatography to obtain a large amount of white solid, which was the intermediate ***H*L-1**: 9-(4-bromobutyl)-9H-carbazole with yield of about 86%.^{1}H-NMR (400MHz, CD_2Cl_2) δ 8.16 (d, J = 7.8 Hz, 1H), 7.50 (m, 2H), 7.28 (dd, J = 7.9, 6.8 Hz, 1H), 4.39 (t, J = 7.2 Hz, 1H), 3.44 (t, J = 6.5 Hz, 1H), 2.07 (m, 1H), 1.95 (m, 1H) ppm. ^{13}C-NMR (400MHz, CD_2Cl_2) δ 140.3, 125.7, 122.8, 120.3, 118.9, 108.7, 42.2, 33.4, 30.4, 27.6 ppm. Elemental analysis for $C_{16}H_{16}BrN$, calcd (%): C 63.59, H 5.34, N 4.62; found (%): C 64.07, H 5.63, N 4.58.

The eluent was selected to be dichloromethane: petroleum ether was 1:8. K_2CO_3 (80 mol, 11.04 g), acetylacetone (60 mmol, 6 ml), a small amount of 18-crown-6 was added to a three-neck round bottom flask containing 100 mL of acetone. 9-(4-bromobutyl)-9H-carbazole (20 mmol, 6.038 g) was dissolved in 60 mL of acetone and placed in a dropping funnel and attached to a three-necked flask. After installing the device, the nitrogen gas was stirred at room temperature for 30 min. After 30 minutes, the temperature was raised to 50 °C, and the 9-(4-bromobutyl)-9H-carbazole solution in the dropping funnel was gradually added dropwise to the three-necked flask, and the mixture was heated to reflux, and the nitrogen was kept for 17 hours. After the reaction is completed, distilled water is added, and the mixture is extracted with a dichloromethane solution, and then steamed, and purified by column chromatography. The eluent is selected to be dichloromethane: petroleum ether is 1:10 to obtain colorless needle crystals which is intermediate ***H*L-2**: 3-(4-(9H-carbazol-9-yl)butyl)pentane-2,4-dione with yield of about 51%. ^{1}H-NMR (400MHz, CD_2Cl_2) δ 8.13 (d, J = 7.8 Hz, 1H), 7.48 (m, 2H), 7.26 (dd, J = 7.4, 6.9 Hz, 1H), 4.35 (t, J = 7.2 Hz, 1H), 3.58 (t, J = 7.1 Hz, 1H), 2.12 (m, 3H), 1.92 (m, 1H), 1.84 (m, 1H), 1.35 (m, 1H) ppm. ^{13}C-NMR (400MHz, CD_2Cl_2) δ 204.0, 140.4, 125.6, 122.7, 120.2, 118.8, 108.7, 68.1, 42.7, 29.2, 28.8, 27.8, 25.3 ppm. Elemental analysis for $C_{21}H_{23}NO_2$, calcd (%): C 78.47, H 7.21, N 4.36; found (%): C 78.67, H 7.40, N 4.37.

The 3-(4-(9H-carbazol-9-yl)butyl)pentane-2,4-dione was added to a flask containing hydrazine hydrate (80%,

14 mL), ethanol (100 mL), kept at 70 °C for 15 h, and the ethanol was evaporated to give a yellow oil. After freezing in a refrigerator for one week, it was taken out, and a white solid was observed. After washing with a diethyl ether solution, it was filtered and dried to give a product: ***H*L** [9-(4-(3,5-dimethyl-1H-pyrazol-4-yl)butyl)-9H-carbazole] yield of about 39%. ^{1}H-NMR (400MHz, CD_2Cl_2) δ 8.13 (d, J = 7.8 Hz, 1H), 7.50 (m, 2H), 7.25 (dd, J = 7.9, 6.7, 1H), 4.35 (t, J = 7.2Hz, 1H), 2.38 (t, J = 7.6 Hz, 1H), 2.13 (s, 3H), 1.90 (m, 1H), 1.56 (m, 1H) ppm. ^{13}C-NMR (400MHz, CD_2Cl_2) δ 141.8, 140.4, 125.6, 122.7, 120.2, 118.7, 115.0, 108.7, 43.0, 28.7, 28.2, 22.8, 10.6 ppm. IR spectrum (KBr, cm^{-1}): 3203 (w), 3147(w), 3089 (w), 3042 (w), 3007 (w), 2928 (m), 2856 (w),1626 (m), 1592 (m), 1482 (m), 1451 (m), 1413 (m), 1383 (w), 1325 (m), 1298 (w), 1238 (m), 1206 (m), 1177 (w), 1153 (m), 1104 (w), 1066 (w), 1020 (w), 1000 (w), 924 (w), 901 (w), 850 (w), 771 (w), 749 (s), 721 (s), 628 (w), 615 (w), 556 (w), 527 (w), 445 (w), 421 (m). Elemental analysis for $C_{21}H_{23}N_3$, calcd (%): C 79.46, H 7.30, N 13.24; found (%): C 79.61, H 7.41, N 13.41.

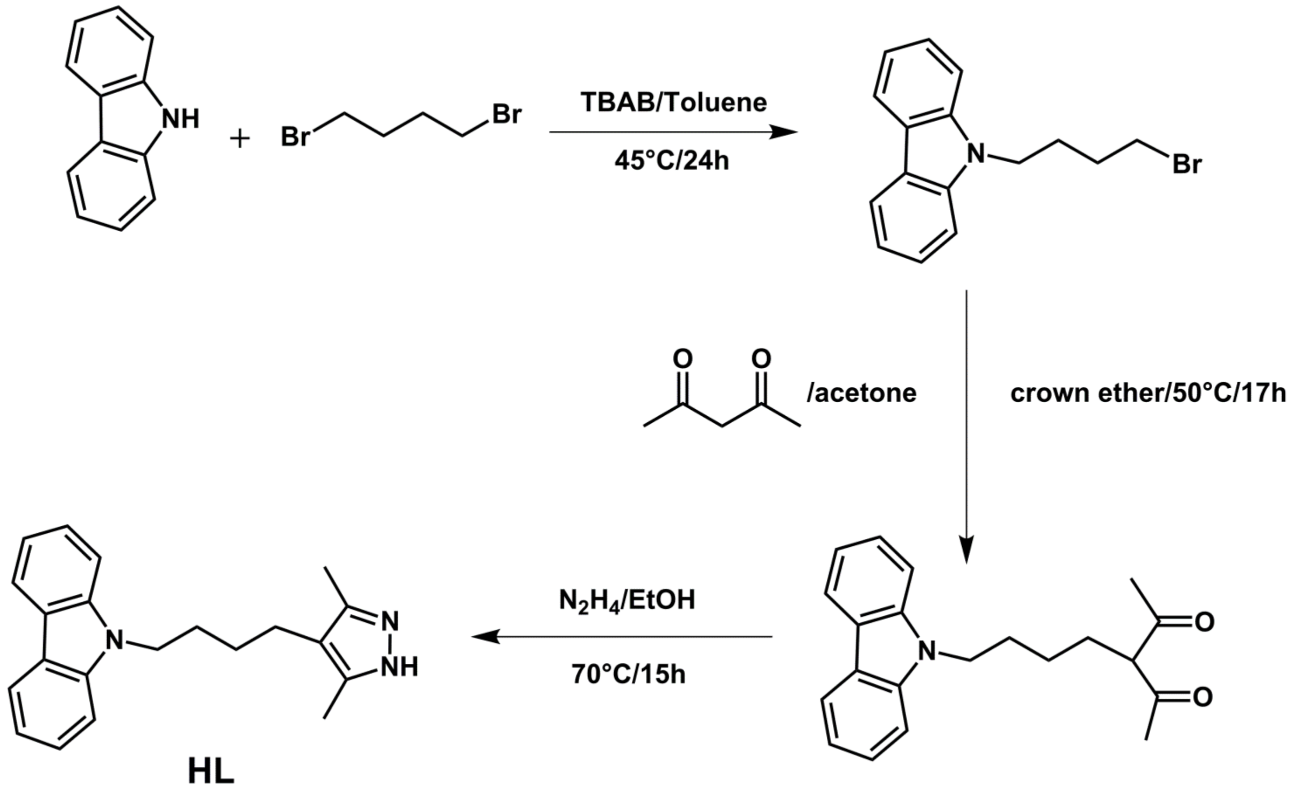

Scheme S1. Synthetic route of ***H*L**.

1a: Cu_2O (4.32 mg, 0.03 mmol), HL (9.51 mg, 0.03 mmol) ,1.5 mL ethanol and 1.5 ml water were added to a hard glass tube with an inner diameter of 8 mm, seal and heat to 140 °C for 72 hours, then It was cooled to room temperature at a rate of 3 °C per hour, filtered and washed with ethanol to give a light yellow block crystal with a yield of about 52%. Elemental analysis for $C_{63}H_{66}N_9Cu_3$, calcd (%): C 66.15, H 6.06, N 10.74; found (%): C 66.38, H 5.84, N 11.06.

1b: Cu_2O (4.22 mg, 0.03 mmol), HL (9.51 mg, 0.03 mmol) and 3 mL ethanol were added to a hard glass tube with an inner diameter of 8 mm, seal and heat to 140 °C for 72 hours, then It was cooled to room temperature at a rate of 3 °C per hour, filtered and washed with ethanol to give a colorless filamentous crystal with a yield of about 70 %. Elemental analysis for $C_{63}H_{66}N_9Cu_3$, calcd (%): C 66.38, H 5.84, N 11.06; found (%):C 66.12, H 5.65, N 10.96.

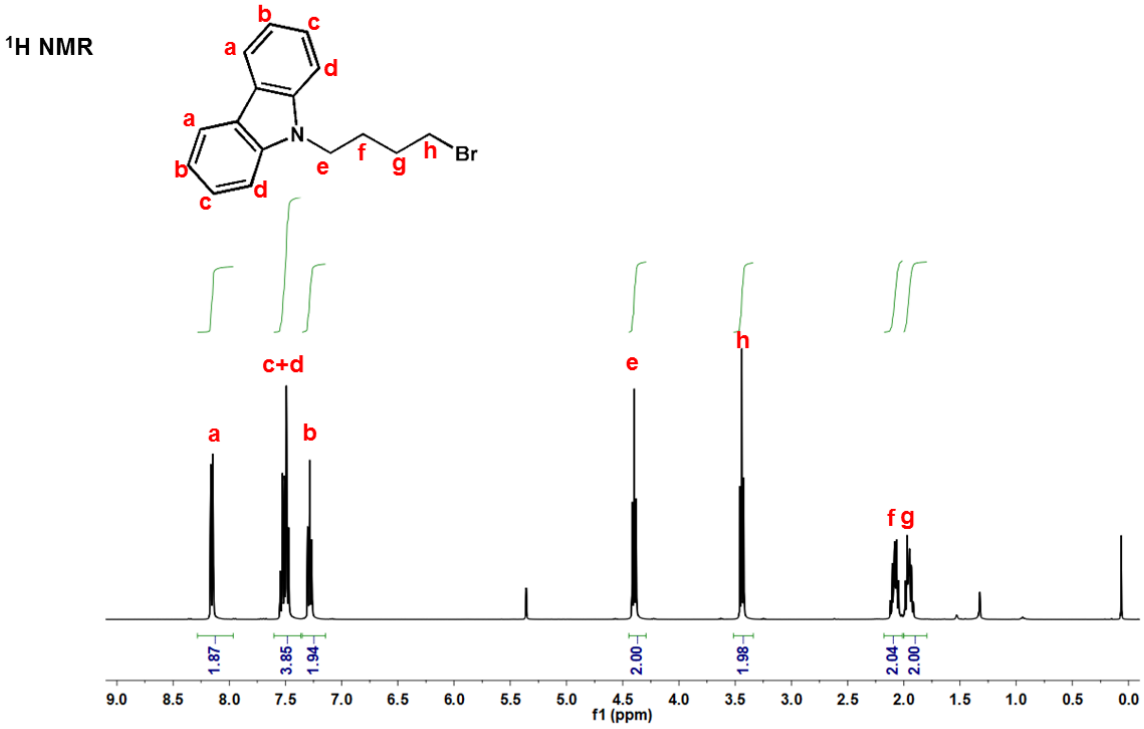

Figure S1. ^{1}H NMR (400MHz, CD_2Cl_2, 298K) spectra of intermediate ***H*L-1**: 9-(4-bromobutyl)-9H-carbazole.

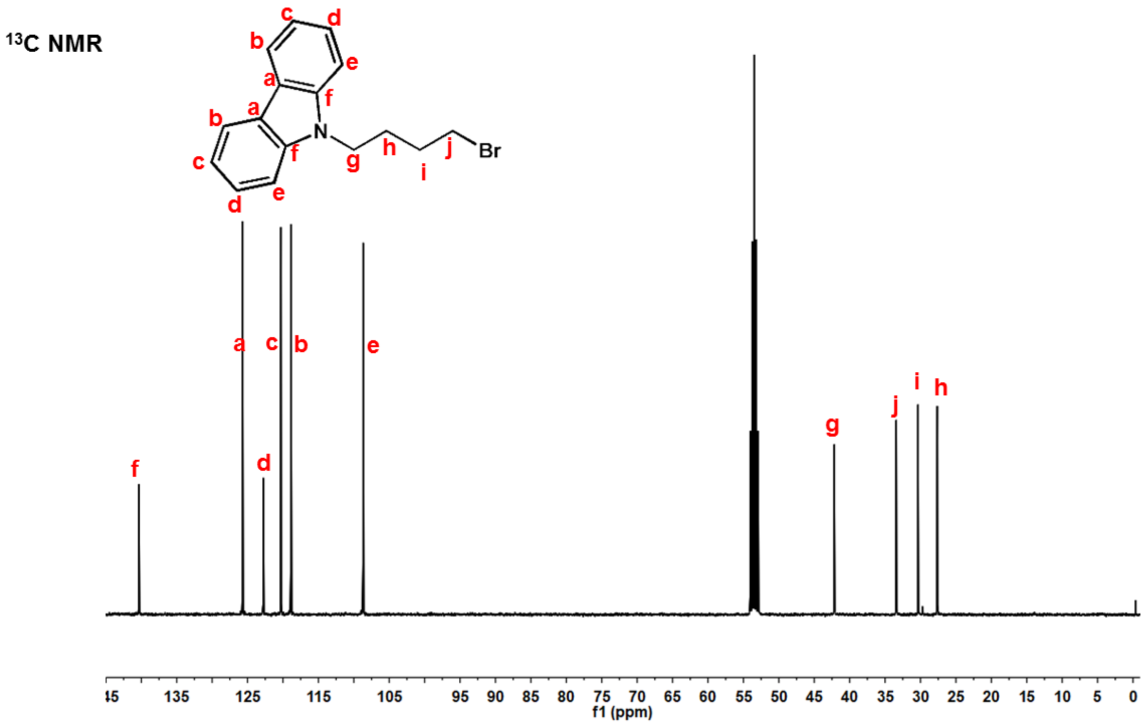

Figure S2. ^{13}C NMR (400MHz, CD_2Cl_2, 298K) spectra of intermediate ***H*L-1**: 9-(4-bromobutyl)-9H-carbazole.

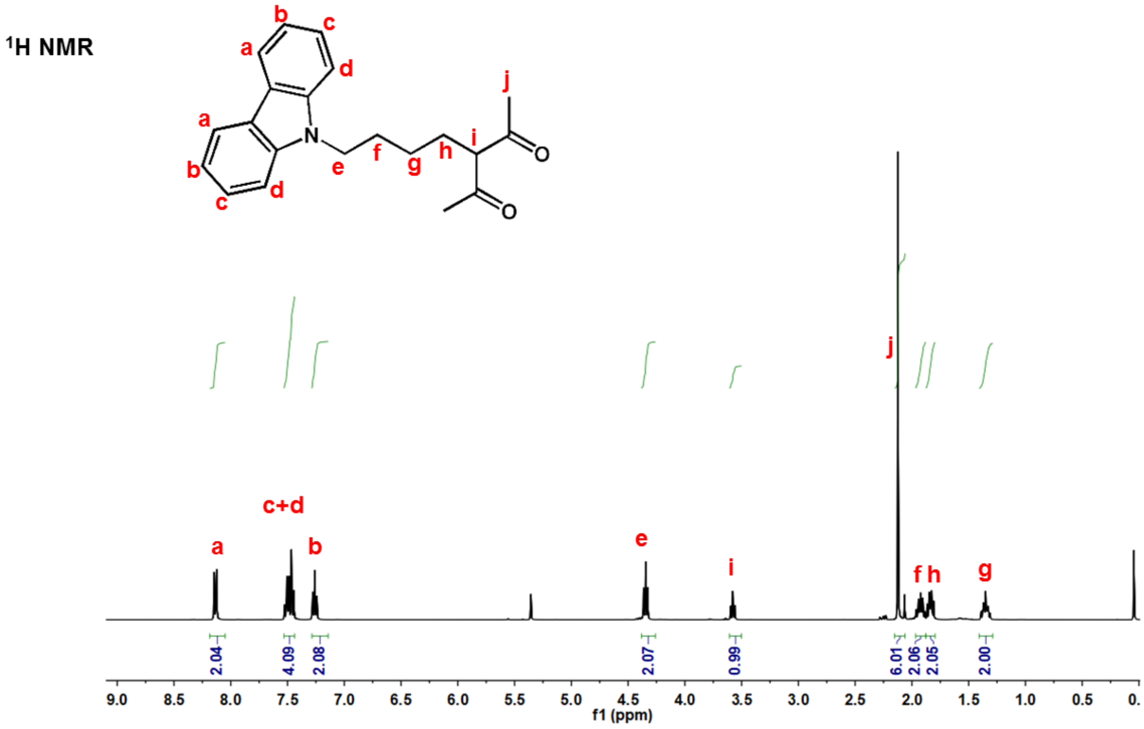

Figure S3. ^{1}H NMR (400MHz, CD_2Cl_2, 298K) spectra of intermediate ***H*L-2**: 3-(4-(9H-carbazol-9-yl)butyl)pentane-2,4-dione.

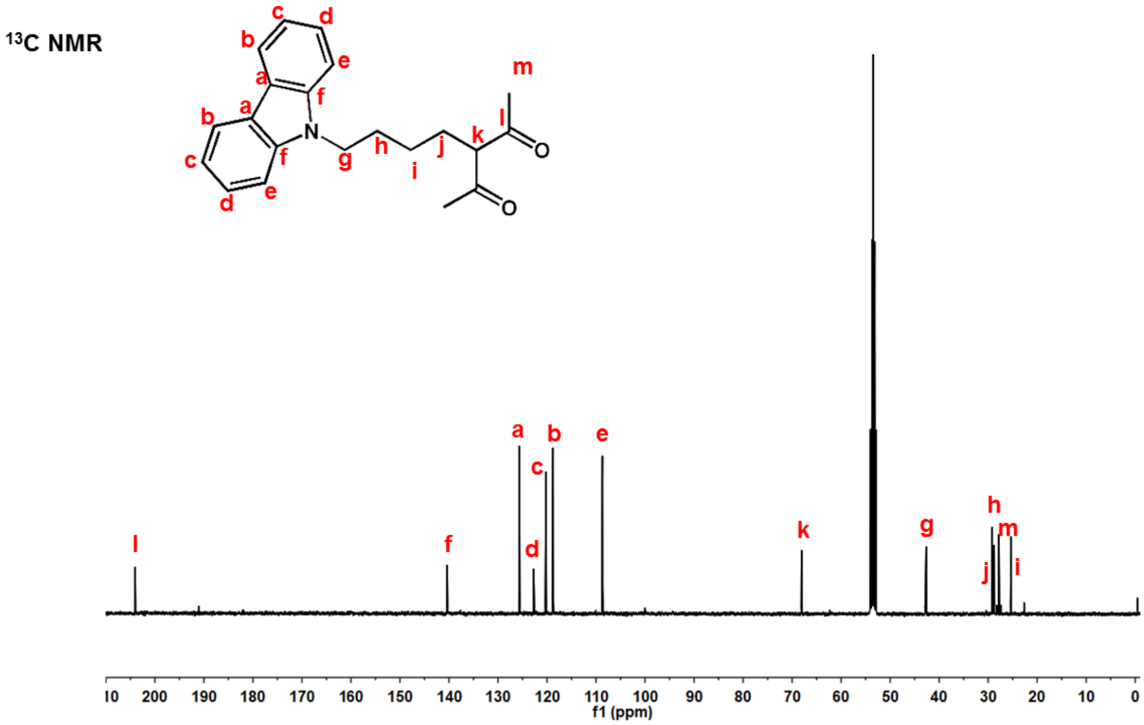

Figure S4. ^{13}C NMR (400MHz, CD_2Cl_2, 298K) spectra of intermediate ***H*L-2**: 3-(4-(9H-carbazol-9-yl)butyl)pentane-2,4-dione.

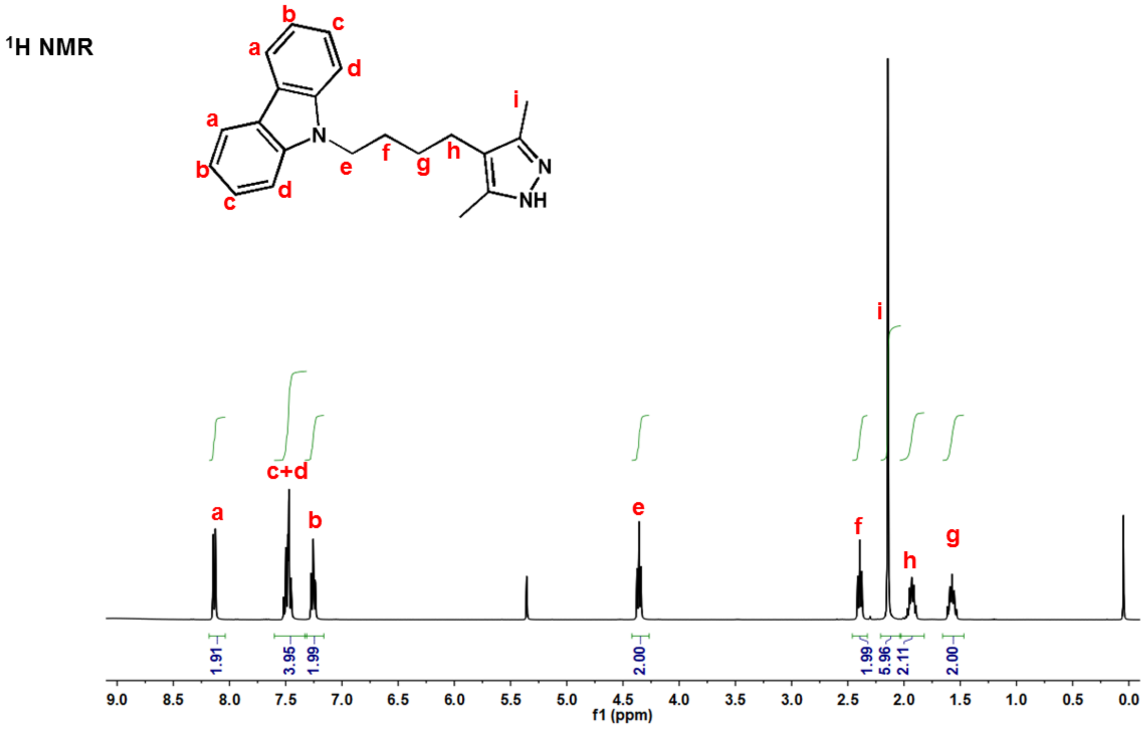

Figure S5. ^{1}H NMR (400MHz, CD_2Cl_2, 298K) spectra of ***H*L**: [9-(4-(3,5-dimethyl-1H-pyrazol-4-yl)butyl)-9H-carbazole].

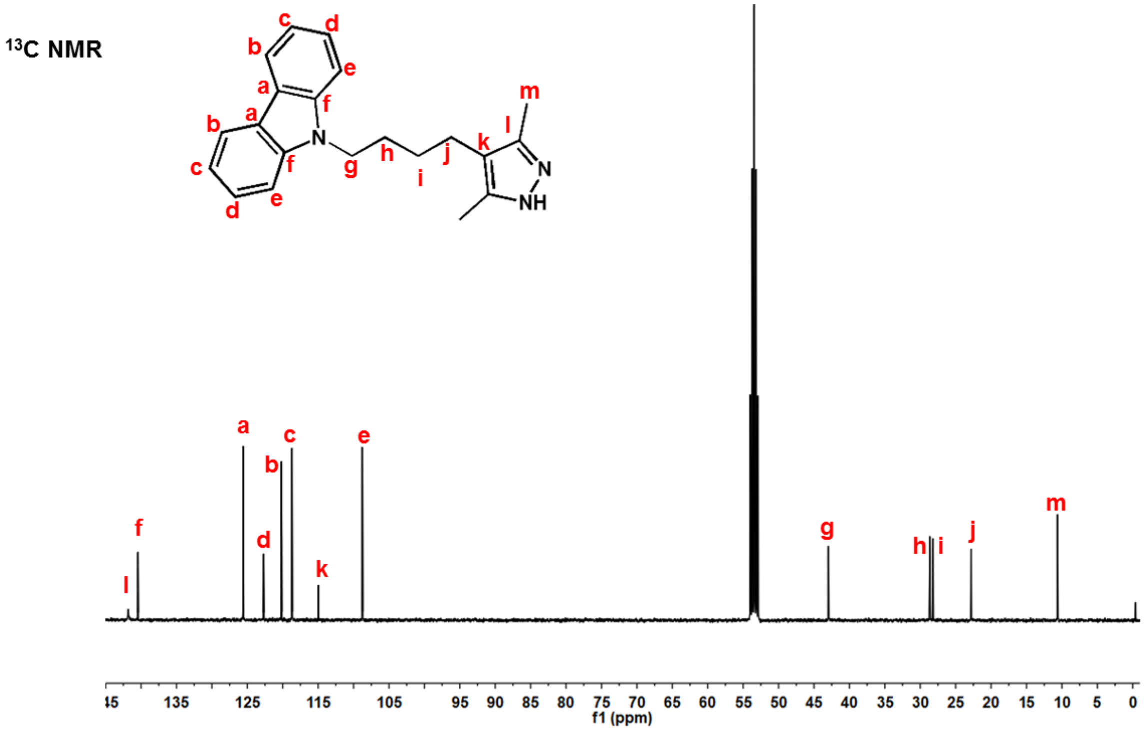

Figure S6. ^{13}C NMR (400MHz, CD_2Cl_2, 298K) spectra of ***H*L**: [9-(4-(3,5-dimethyl-1H-pyrazol-4-yl)butyl)-9H-carbazole].

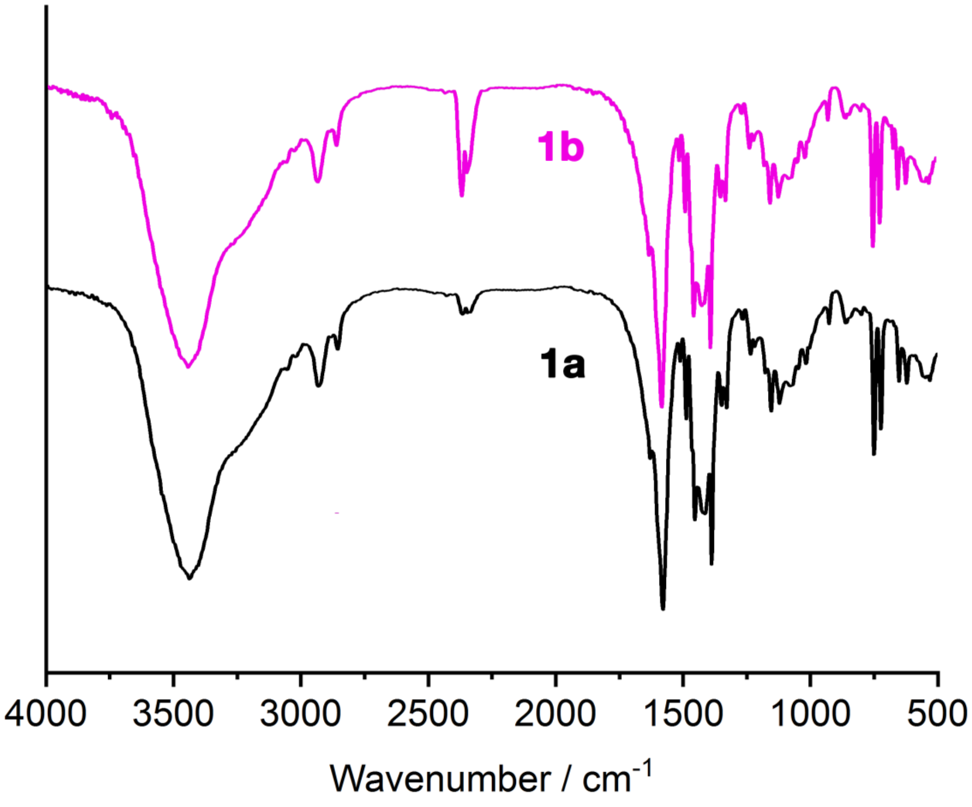

Figure S7. FT-IR spectra of **1a** and **1b**.

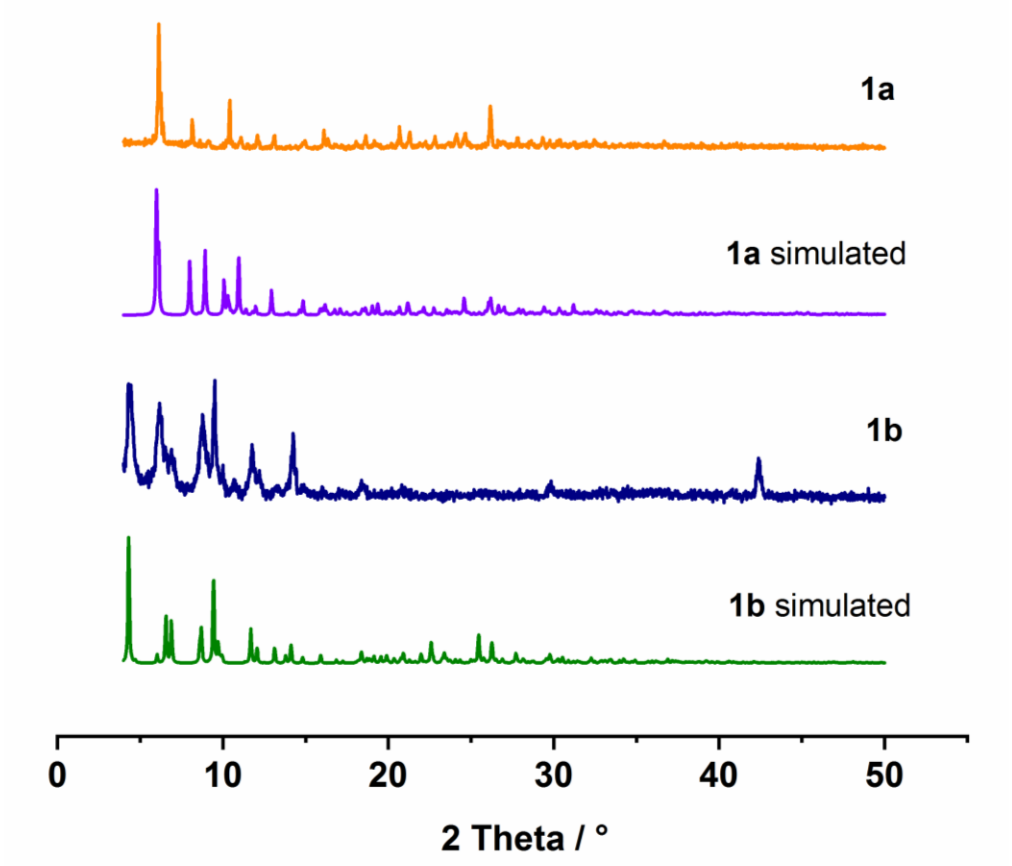

Figure S8. PXRD patterns for simulated, as-synthesized sample of **1a** and **1b**.

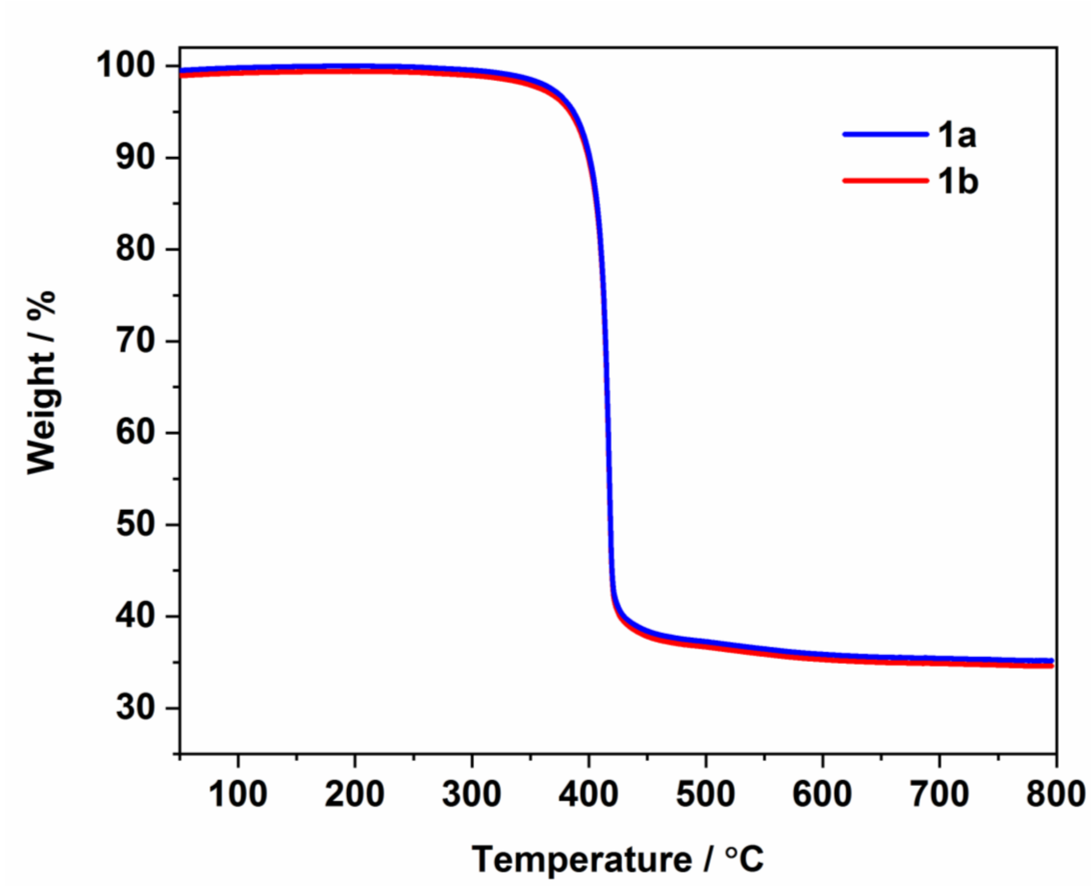

Figure S9. Thermogravimetric analysis (TGA) curves of **1a** and **1b**.

Crystallography study

Single-crystal X-ray diffraction data for **1a** and **1b** were collected *via* an Oxford Cryo stream system on a XtaLAB PRO MM007-DW diffractometer system equipped with a RA-Micro7HF-MR-DW(Cu/Mo) X-ray generator and Pilatus3R-200K-A detector (Rigaku, Japan, Cu Kα, λ = 1.54178 Å). The numerical absorption corrections were applied using the program of ABSCOR. The data set temperatures were 296 K and 100 K for **1a** and 298 K for **1b**. For **1a**, the structures were solved using direct methods, which yielded the positions of all non-hydrogen atoms. These were refined first isotropically and then anisotropically. All the hydrogen atoms of the ligands were placed in calculated positions with fixed isotropic thermal parameters and included in the structure factor calculations in the final stage of full-matrix least-squares refinement.

For **1b**, low quality of crystal structure of sample is due to poor data and disorder problem of the carbazole ring. The cell parameters and atomic position of CTC rings and heavy atom were generated from SCXRD data by Patterson method.

All calculations were performed using the SHELXTL system of computer programs.[1,2] Crystal data and structure refinement parameters are summarized in Table S1. Selected bond lengths and angles are given in Table S2.

Table S1. Summary of crystal data and structure refinement parameters for **1a** and **1b.**

sample	**1a**	**1a**	**1b**
Empirical formula	$C_{63}H_{66}N_9Cu_3$	$C_{63}H_{66}N_9Cu_3$	$C_{63}H_{66}N_9Cu_3$
CCDC No.	2043667	2043668	2043666
Formula weight	1137.33	1139.90	1139.90
Temperature (K)	100	296	298
Crystal system	trigonal	monoclinic	monoclinic
Space group	*Pc*	$P2_1/c$	$P\bar{1}$
a (Å)	11.4020(2)	11.52641(11)	9.7333(10)
b (Å)	28.8860(5)	29.1085(3)	27.941(2)
c (Å)	17.7029(3)	17.85854(17)	42.675(4)
α (°)	90	90	75.096(7)
β (°)	104.146(2)	103.9899(10)	91.180(2)
γ (°)	90	90	83.457(7)
Volume (Å^3)	5656.66(7)	5814.11(10)	11121.3(18)
Z	4	4	8
$D_{calcd.}$ (g/cm^3)	1.339	1.302	1.364
F (000)	2376.0	2376.0	4752.0
μ (mm^{-1})	1.674	1.629	1.186
2θ range for data collection (°)	5.99 - 155.782	7.932 - 155.944	5.766 - 56.126
reflns. collected	22991	61630	72260
Data / restraints / parameters	23733 / 2 / 1363	12209 / 0 / 736	12181 / 3 / 462
R_{int}	0.0158	0.0208	0.3024
Goodness-of-fit on F^2	1.062	1.054	1.119
R_1 [I>2σ(I)] ^{a}, wR_2 [I>2σ(I)] ^{b}	R_1 = 0.0990, wR_2 = 0.2510	R_1 = 0.0573, wR_2 = 0.1758	R_1 = 0.1609, wR_2 = 0.2386

$^aR_1 = \sum|F_o| - |F_c||/\sum|F_o|$. ^{b} $wR_2 = \{[\sum w(F_o^2-F_c^2)^2]/\sum[w(F_o^2)^2]\}^{1/2}$; $w = 1/[\sigma^2(F_o^2) + (aP)^2 + bP]$, where $P = [\max(F_o^2,0) + 2F_c^2]/3$ for all data

Table S2. Comparison of selected bond length (Å) and bond angel (°) for **1a** and **1b**.

**Bond length/angle**	**1a-100 K**	**1a-296 K**	**1b-298 K**
Cu-N	1.853(4),	1.856(4),	1.75(2),
	1.852(3),	1.857(4),	1.90(3),
	1.860(3),	1.848(3),	1.87(3),
	1.853(3),	1.853(3),	1.77(3),
	1.847(3),	1.842(3),	1.82(3),
	1.858(3)	1.842(3),	1.83(3)
Cu-Cu	3.211(2),	3.183(5),	3.17(2),
(intra-trimer)	3.196(4),	3.207(8),	3.18(9),
	3.255(4)	3.212(4)	3.25(2)
Cu-Cu	2.8542(5),	2.9911(7),	3.756(3),
(inter-trimer)	2.9083(6)	2.9912(7)	3.942(2)
N-Cu-N	170.22,	175.01,	176.35,
(intra-trimer)	175.46,	171.51,	176.74,
	175.53	175.31	178.81

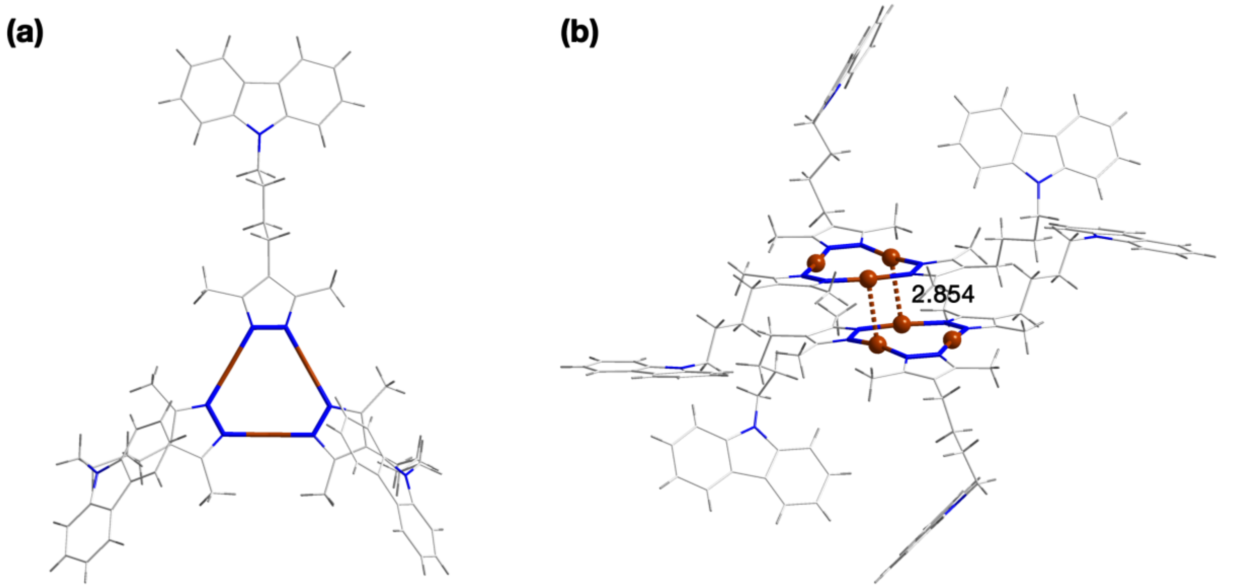

Figure S10. (a) Asymmetric unit of **1a** crystal structure at 100K. (b) Packing diagram of **1a**. Colour representations: dark red, Cu; blue, nitrogen; grey, carbon.

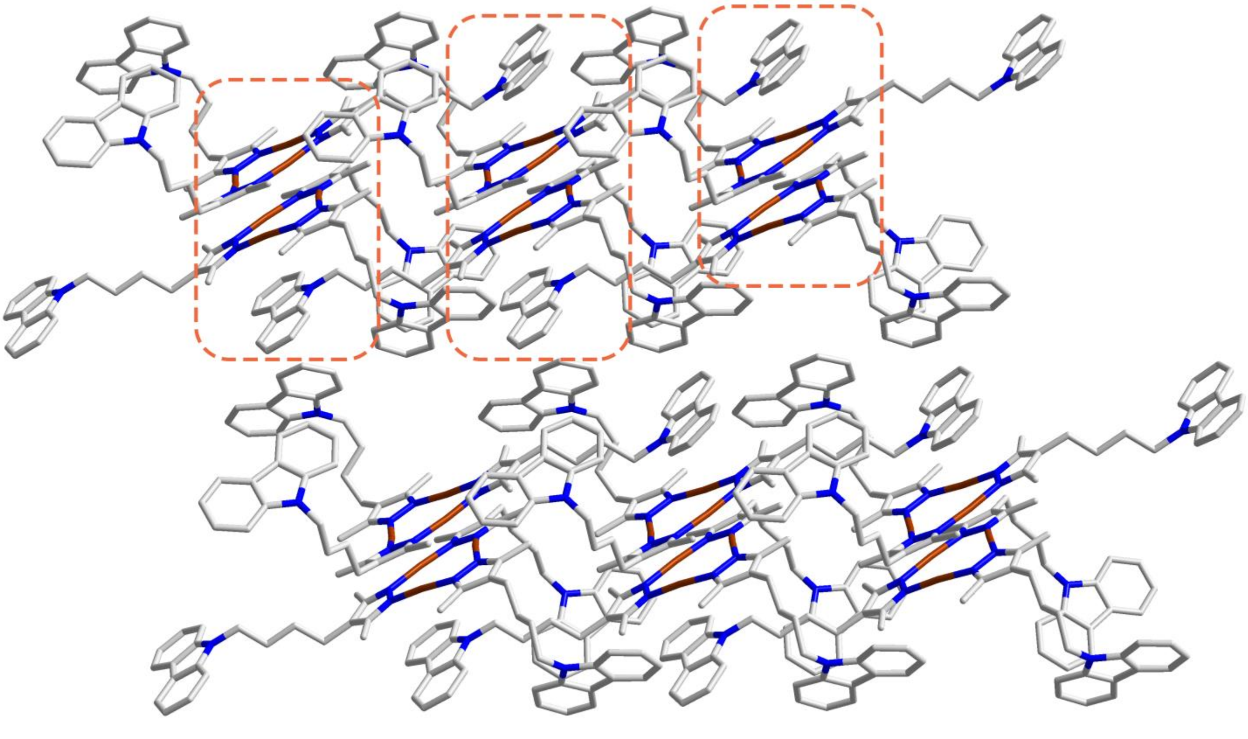

Figure S11. Top viewed packing diagram of **1a**. The CTC packing area and carbazole packing area are highlighted. Colour representations: dark red, Cu; blue, nitrogen; grey, carbon.

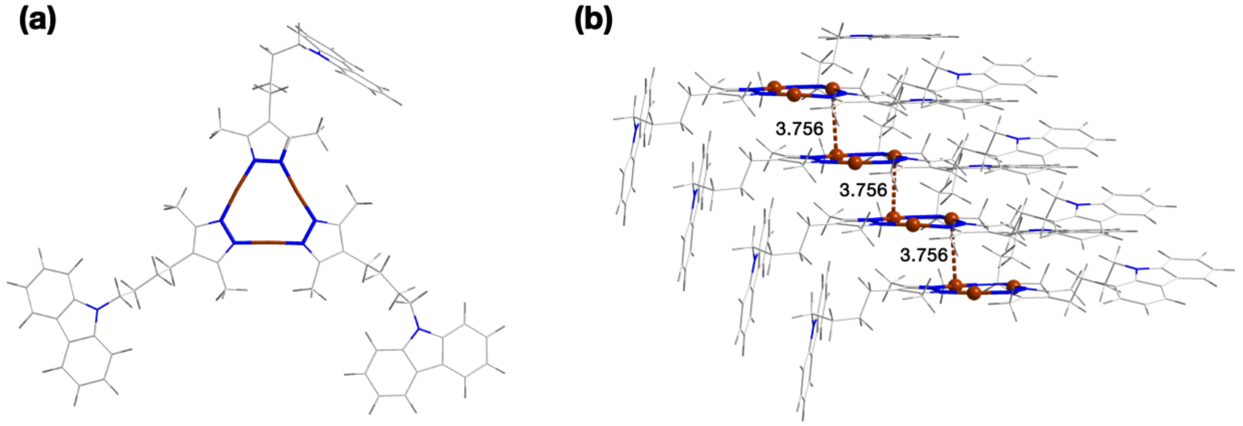

Figure S12. (a) Asymmetric unit of **1b** crystal structure at 298K. (b) Dimeric diagram of **1b**. Colour representations: dark red, Cu; blue, nitrogen; grey, carbon.

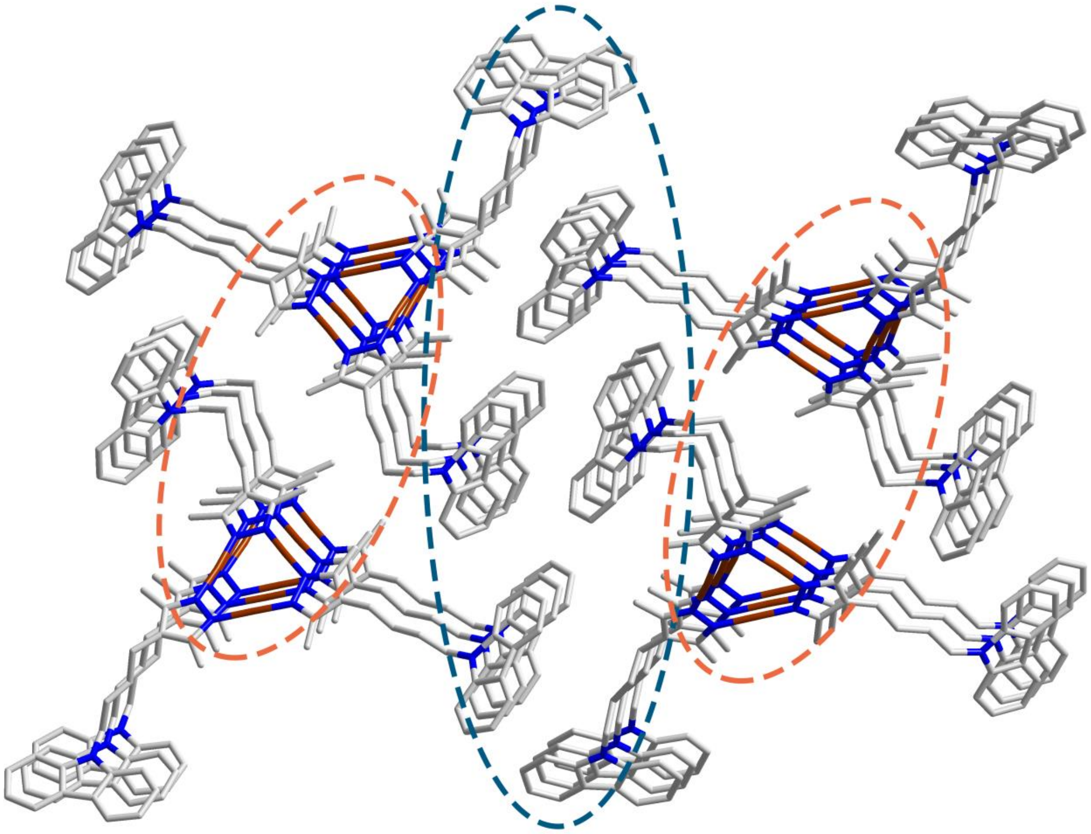

Figure S13. Packing diagram of **1b**. Colour representations: dark red, Cu; blue, nitrogen; grey, carbon.

Photoluminescence Measurement

Steady-state photoluminescence spectra and lifetime measurements were performed by using a single-photon counting spectrometer on an Edinburgh FLS920 spectrometer equipped with a continuous Xe900 xenon lamp, a μF900 μs flash lamp. A closed helium cycle cryostat (Advanced Research Systems) was used for low-temperature luminescence experiments. The absolute photoluminescence quantum yields were measured by employing Hamamatsu C11347-01 absolute PL quantum yield spectrometer under both room temperature and liquid nitrogen. Pressure-dependent photoluminescence spectra measurements on solid samples in silicone oil were performed with a diamond-anvil cell (DAC, High-Pressure Diamond Optics). The ruby R1 fluorescence line method was used to calibrate the hydrostatic pressure inside the gasketed cell. Excitation sources were a 355 nm LED laser for the luminescence experiments. All pressure-induced phenomena reported here are reversible upon gradual release of external pressure.

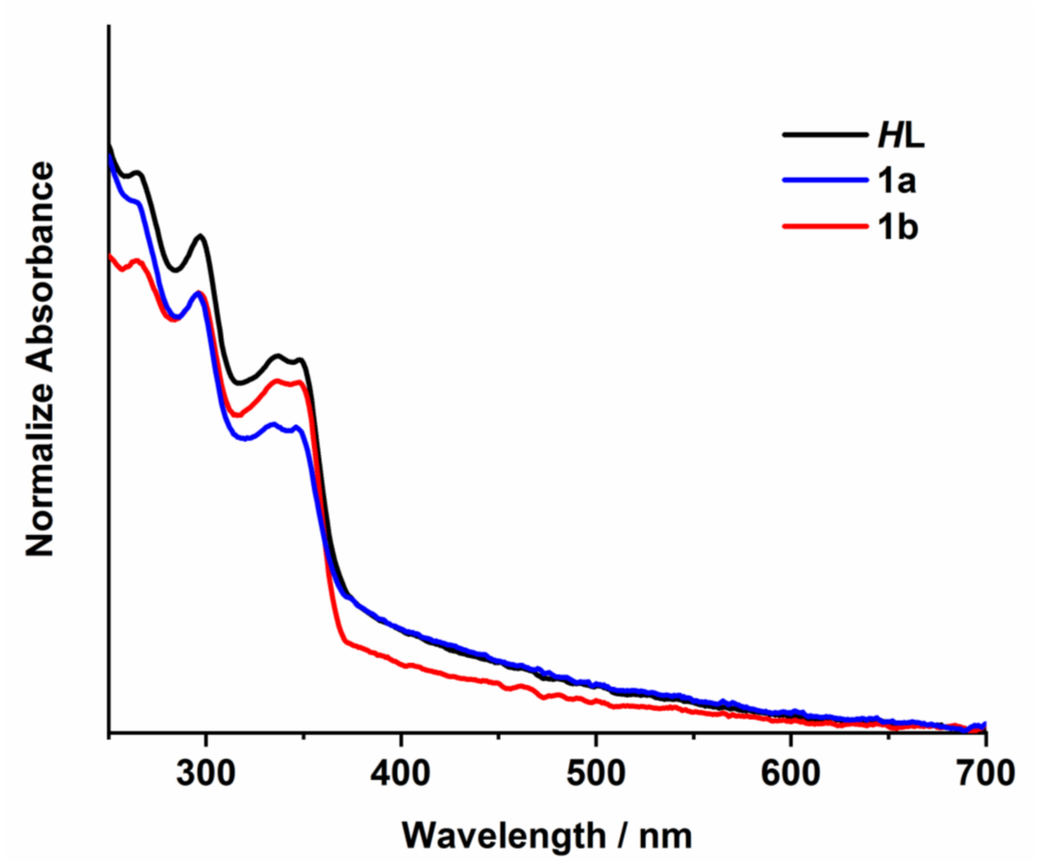

Figure S14. Solid-state UV-vis spectra of *H*L ligand, **1a** and **1b**.

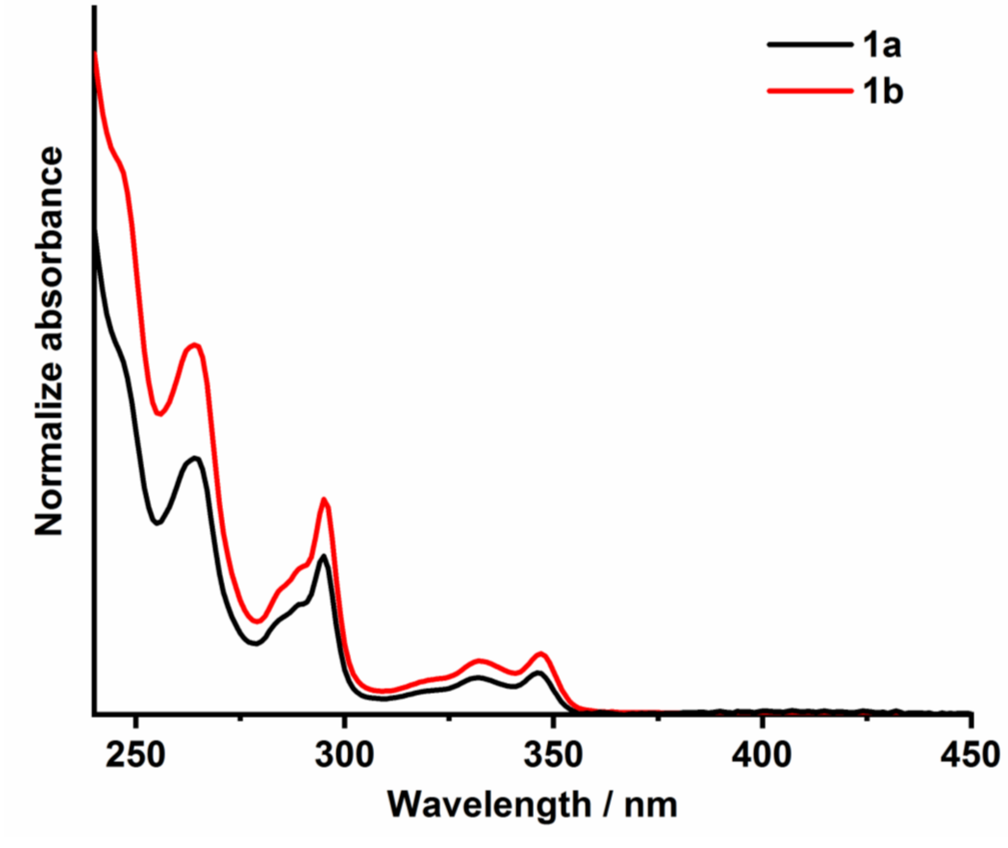

Figure S15. UV-vis spectra **1a** and **1b** in CH_2Cl_2 solution.

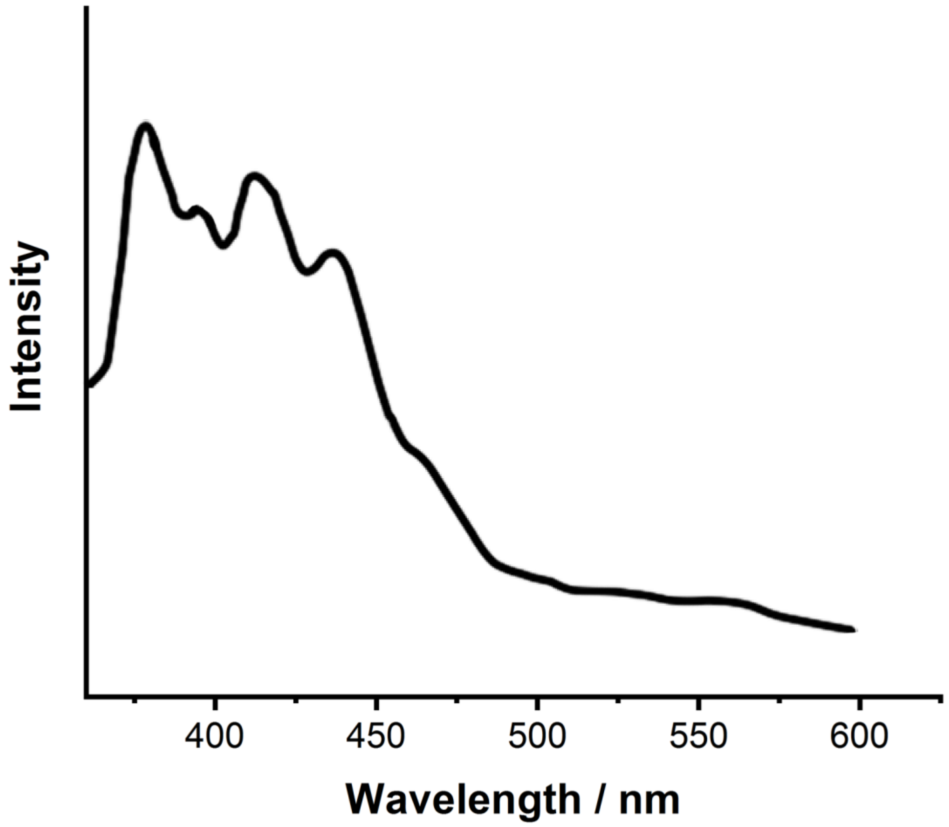

Figure S16. Solid-state emission spectra of *H*L ligand (Ex=360nm).

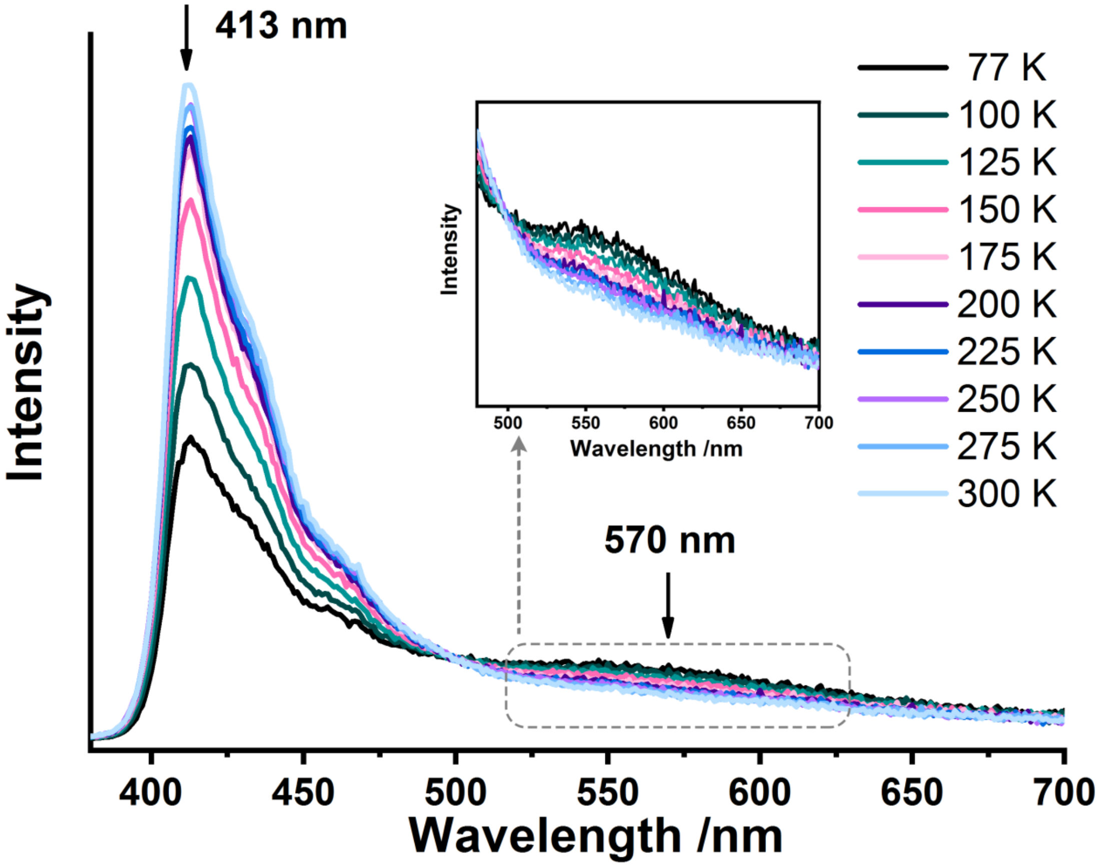

Figure S17. Varied-temperature solid-state emission spectra of *H*L ligand (Ex=360nm).

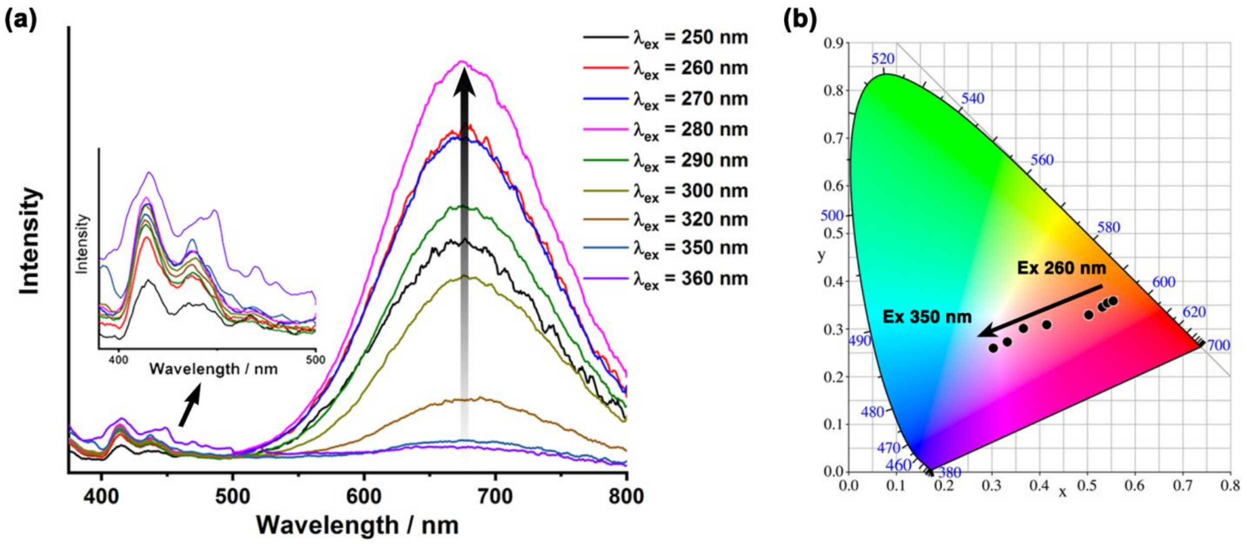

Figure S18. Varied-excitation wavelength solid-state emission spectra (a) and emission colour profiles in CIE-1931 chromaticity diagram (b) of **1a** at room temperature.

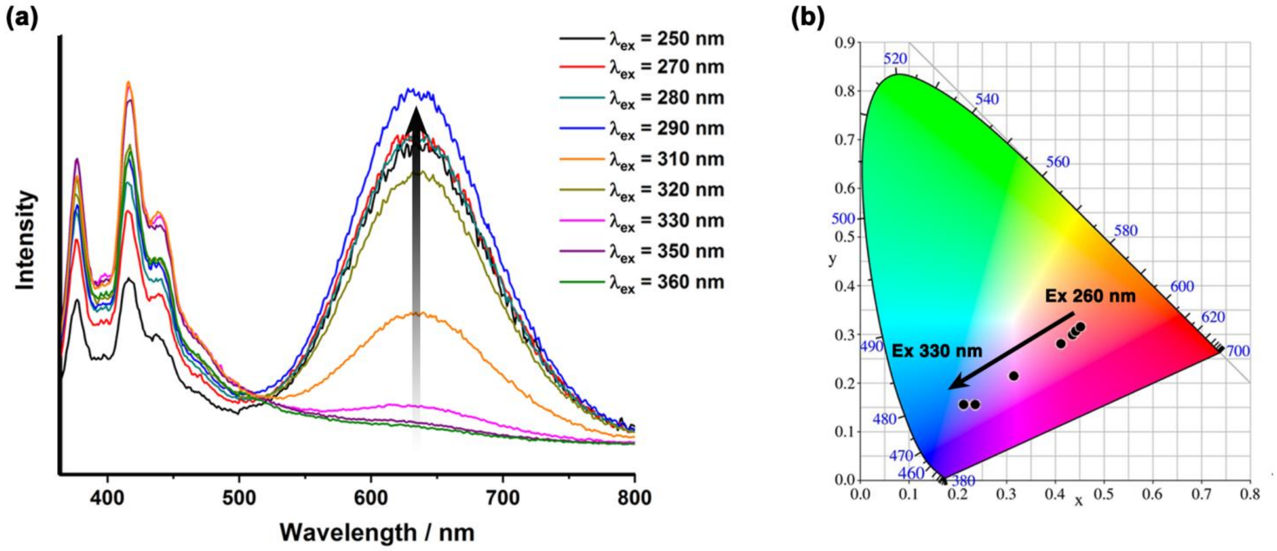

Figure S19. Varied-excitation wavelength solid-state emission spectra (a) and emission colour profiles in CIE-1931 chromaticity diagram (b) of **1b** at room temperature.

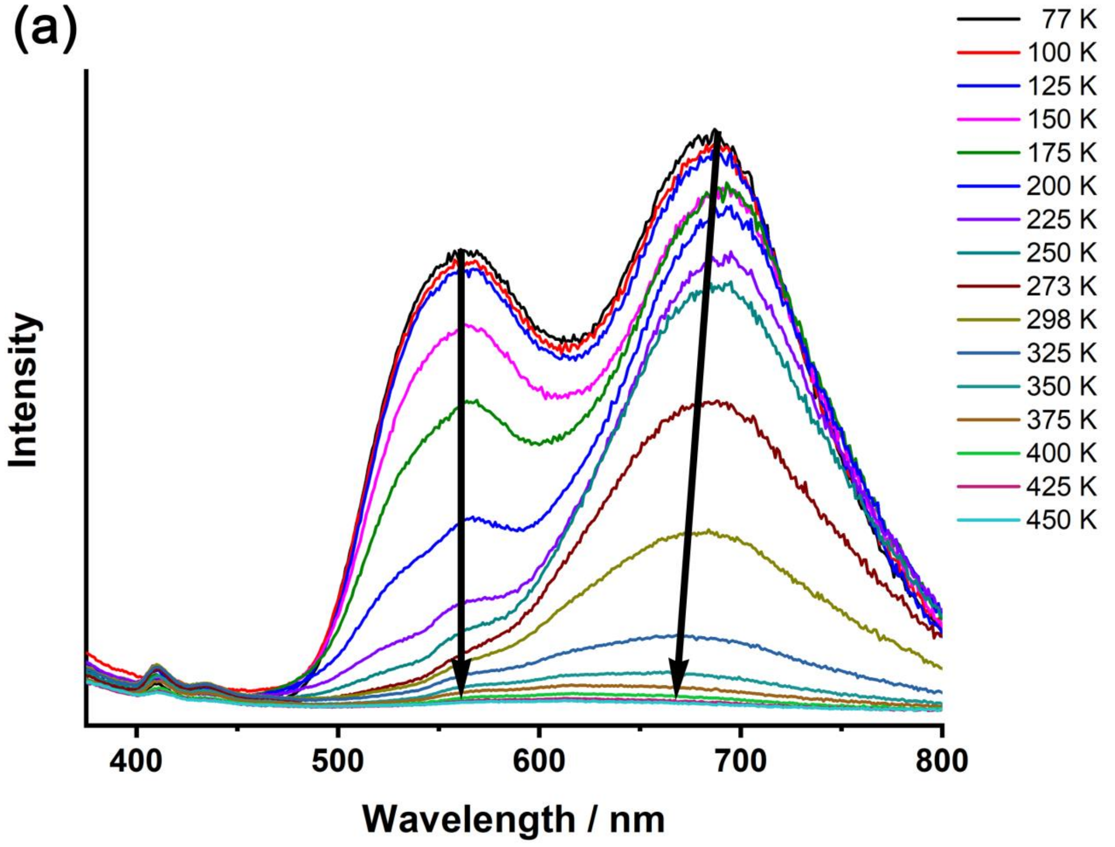

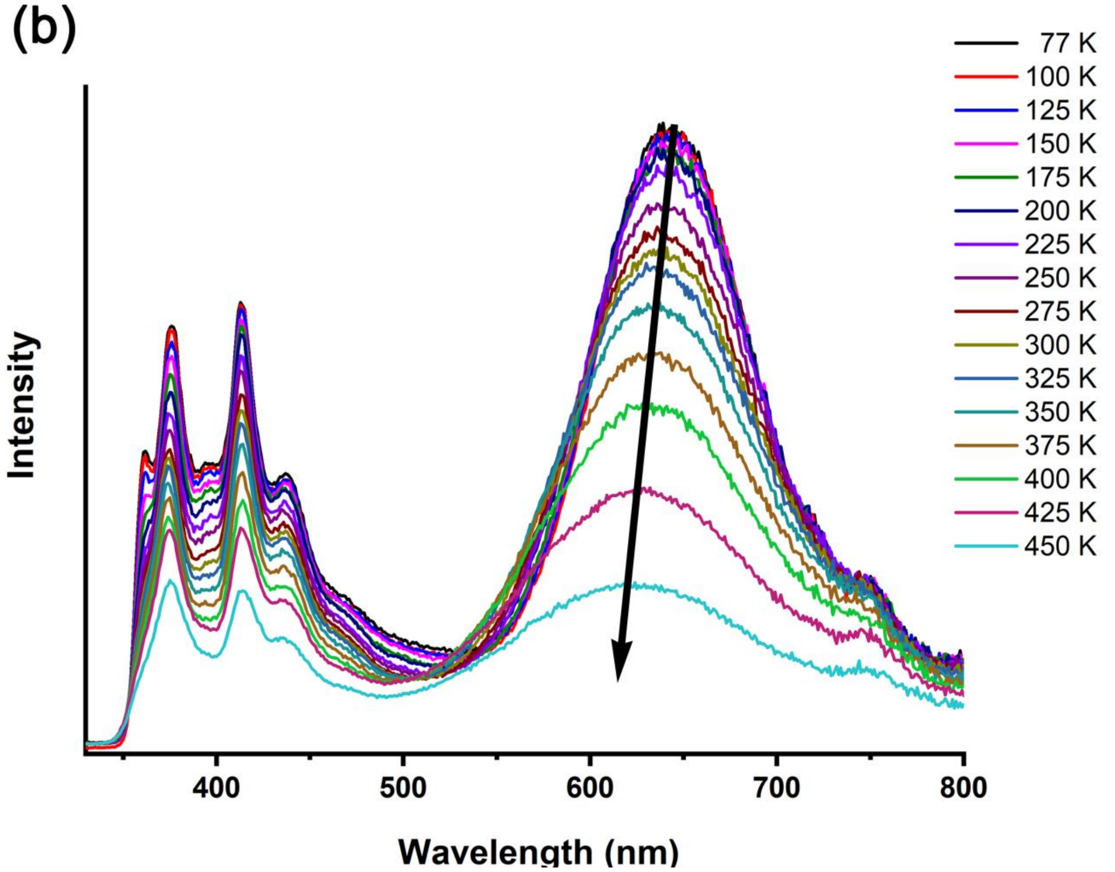

Figure S20. Varied-temperature solid-state emission spectra of (a) **1a** (Ex = 280 nm) and (b) **1b** (Ex = 280 nm).

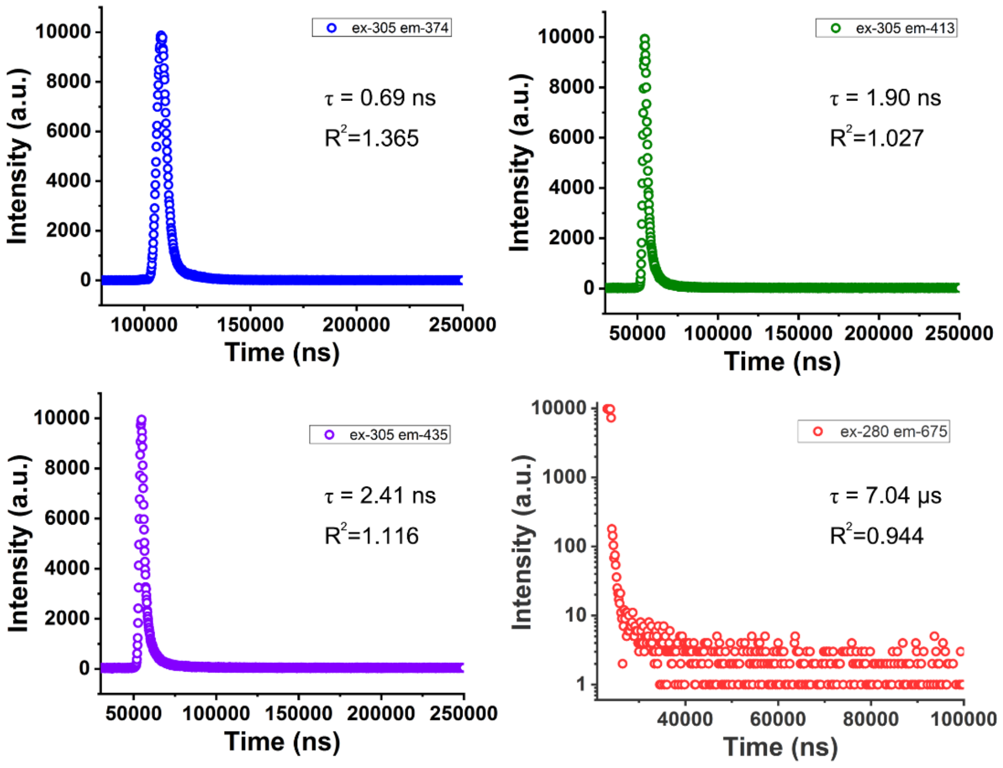

Figure **S21**. Emission decay profiles of **1a** in the solid state.

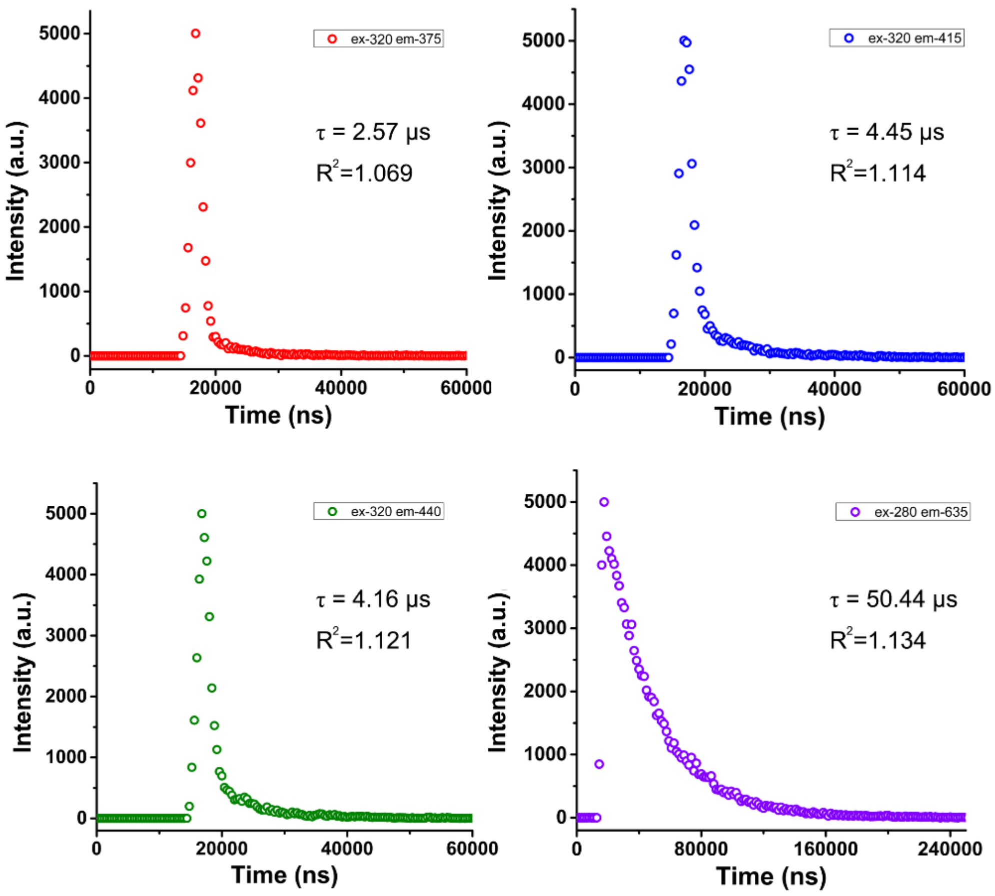

Figure **S22**. Emission decay profiles of **1b** in the solid state.

Table S3. Photophysical data for **1a** and **1b** in the solid state.

	λ_{ex}/nm	λ_{em}/nm	τ_{av}/ns	Φ_{PL} [a]
**1a**	305	374	0.69	
	305	413	1.90	8.3% [b]
	305	435	2.41	28.9% [c]
	280	675	7.04E+03	
**1b**	320	375	2.57E+03	
	320	415	4.45E+03	33.1% [b]
	320	440	4.16E+03	54.9% [c]
	280	635	5.04E+04	

$\tau_{av} = \Sigma(A_i\tau_i)$. [a] Absolute quantum yield. [b] 298K. [c] 77K.

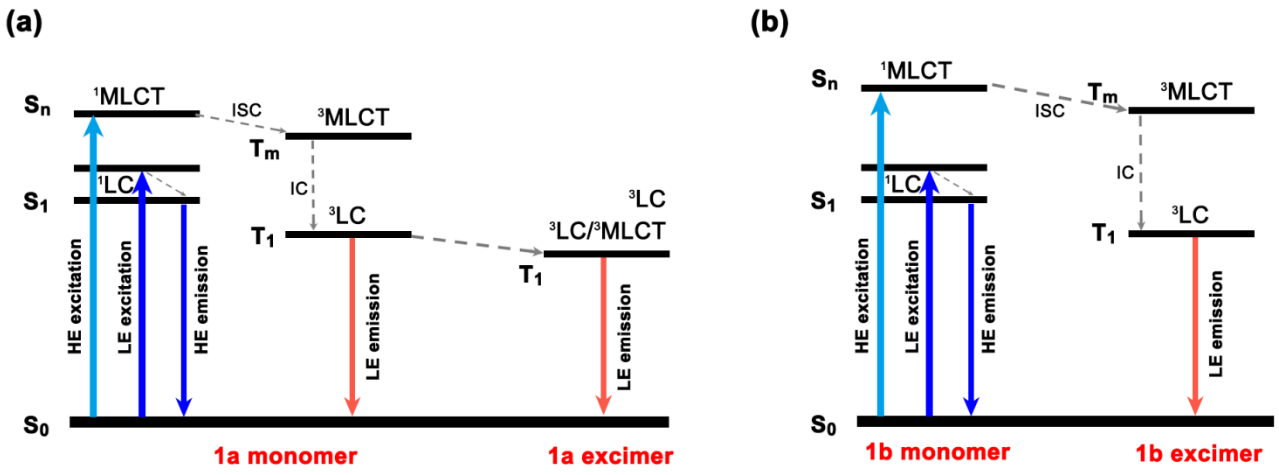

Figure S23. Proposed jablonski diagrams for (a) **1a** and (b) **1b.**

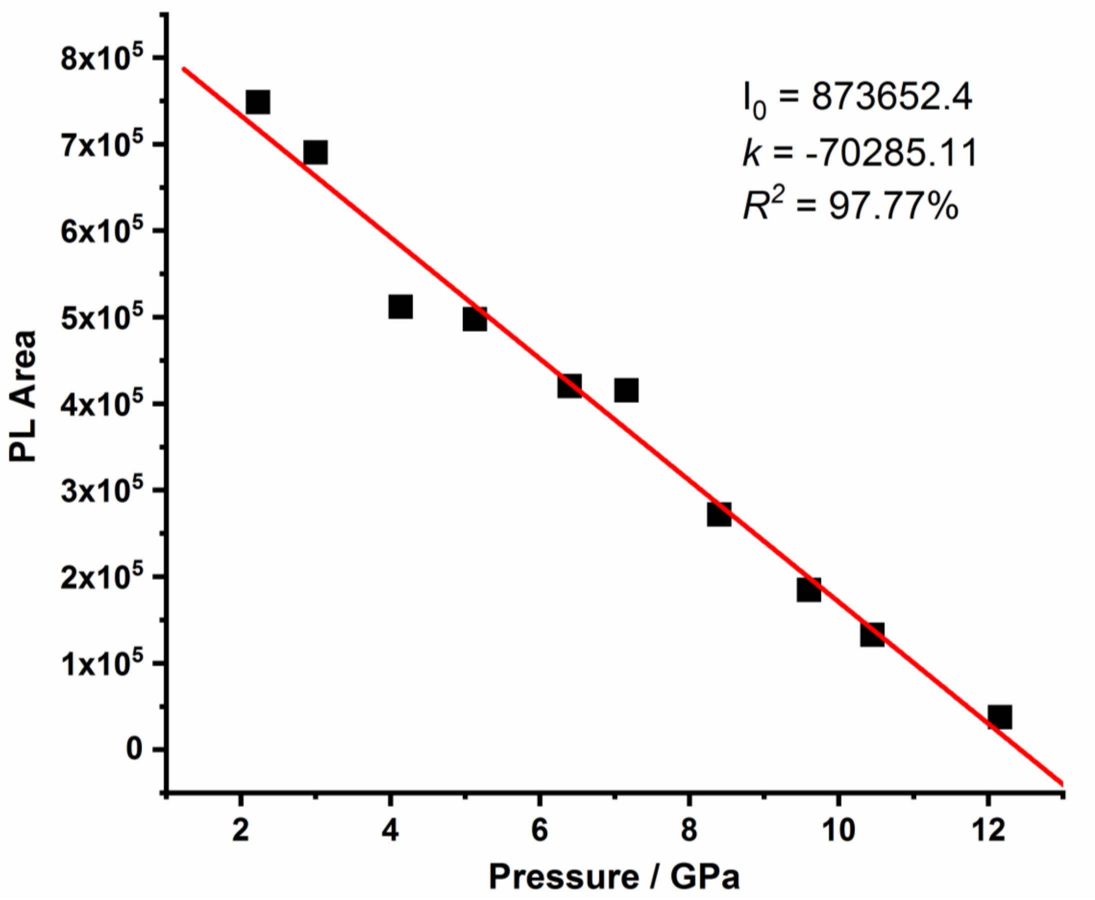

Figure S24. The linear fitting between the external pressure and photoluminescence (PL) integrated area of **1a** in pressure range 2.23 – 12.16 GPa.

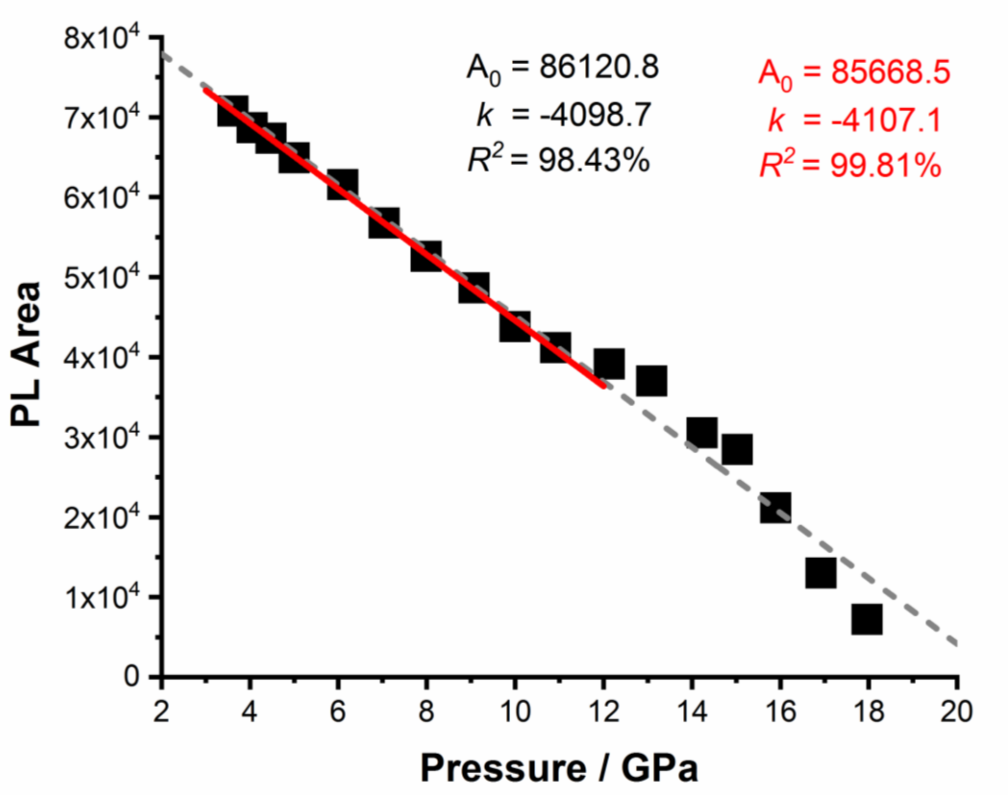

Figure S25. The linear fitting between the external pressure and photoluminescence (PL) integrated area of **1b** in pressure range 3.62 – 17.96 GPa.

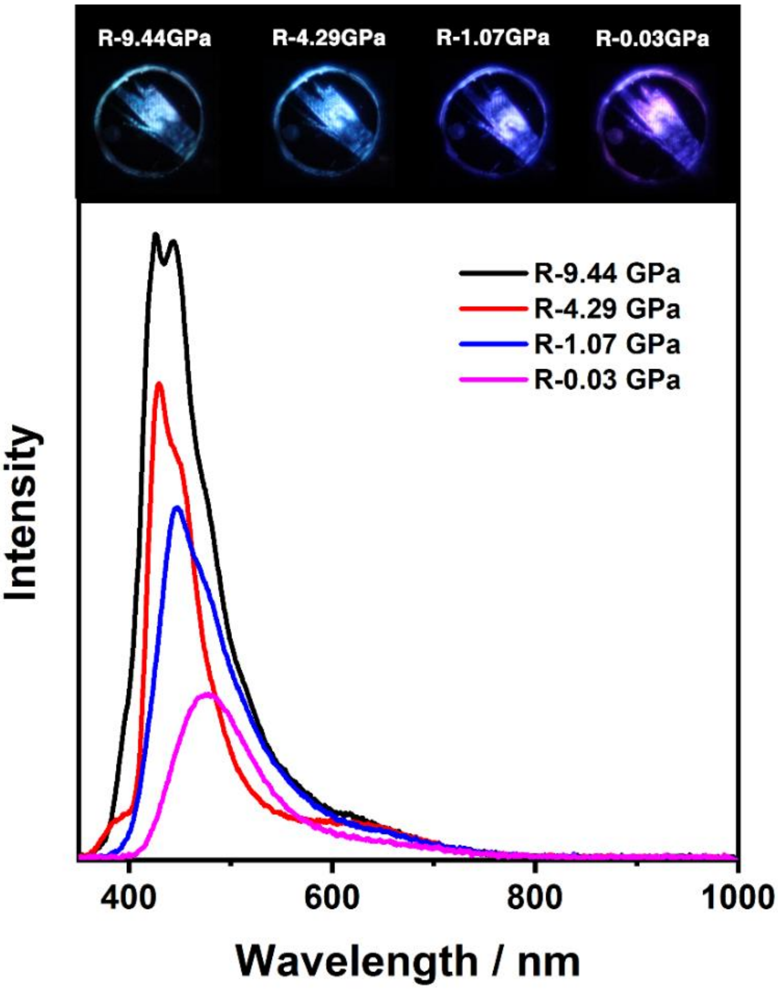

Figure S26. PL spectra and photographs of **1b** in pressure relief from 9.44 GPa to 0.00 GPa.

Computational Details and results

The initial models of **1a** and **1b** were taken from the X-ray crystallography data using one set of disorder structure. Geometry optimization was achieved by density functional theory (DFT) of GGA-PBE method[3] at Gamma point in Cambridge Serial Total Energy Package (CASTEP) modules in Materials Studio 2018 program[4]. The plane wave cut-off energy was 326.5 eV and OTFG ultrasoft pseudopotentials were used for all atoms. Then, the external pressure of 2.5 GPa was applied into the geometry optimization and lattice relaxation of **1a**. The geometry optimization and lattice relaxation of **1b** were done under the external pressure from 4 GPa and 10 GPa. The external pressure is the same in the components of the three directions of x, y and z. The ground state (S_0) geometries of monomer and dimer models of **1a** and **1b** were taken from the CASTEP optimized structure in unit cell at atmospheric pressure without further calculations.

Density functional theory (DFT) and time-dependent density functional theory (TDDFT) were performed by employing PBE0 functional to gain the related electronic excitation information of **1a** and **1b** in Gaussian 09 program[5]. The effective core potential (ECP) of LanL2DZ[6] basis set for Cu atom and 6-31G(d)[7] basis set for other non-metal atoms were utilized. TDPBE0[8] was adopted for geometry optimizations of the lowest singlet (S_1) and triplet excited state (T_1) of monomeric/dimeric **1a** and **1b** in vacuo at normal pressure. The T_1 geometry of **1a [**dimer (b)**]** at 2.5 GPa was constrained optimized based on the CASTEP result at 2.5 GPa by TDPBE0. The vertical excitation energy and corresponding electron transitions as well as the frontier molecular orbital (FMO) analysis was based on the geometry of S_0, S_1 and T_1. The natural transition orbitals (NTOs) were analyzed here to better study the electron transition information. The vertical excitation energy and the corresponding FMO in compression of **1b** were calculated with the same theoretical level based on dimer model from the CASTEP optimized results directly. The NTOs were generated by Multiwfn 3.6 software[9] and major contributions were displayed.

Table S4. TDDFT results of selected singlet-singlet excited state (ES) for **1a** or **1b** in S_0 geometry. *f* refers to the oscillator strength in calculation, major natural transition orbitals (NTO) were demonstrated.

ES	E /eV	λ /nm	*f*	NTO (hole-electron)	Assignment
S_1	4.098	302.7	0.0375	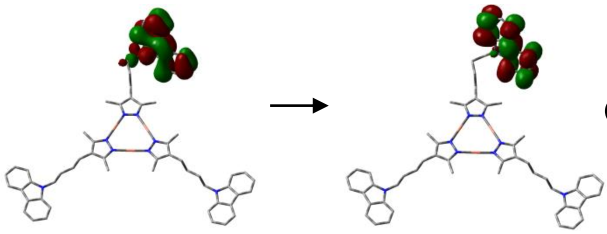 (91%)	^{1}LC
S_2	4.100	302.4	0.0700	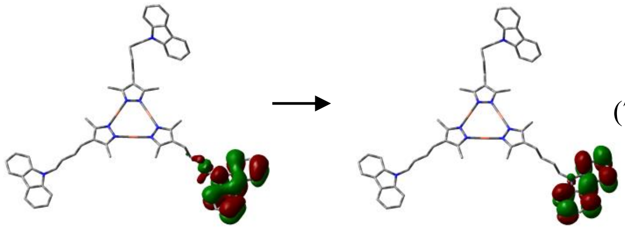 (76%)	^{1}LC
				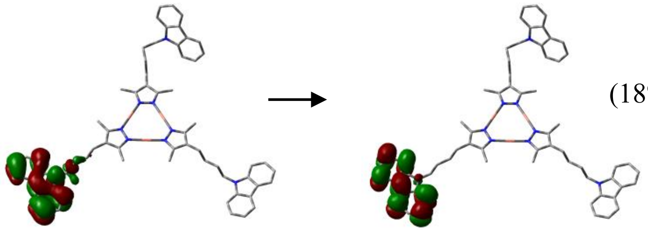 (18%)	
S_7	4.612	268.8	0.0479	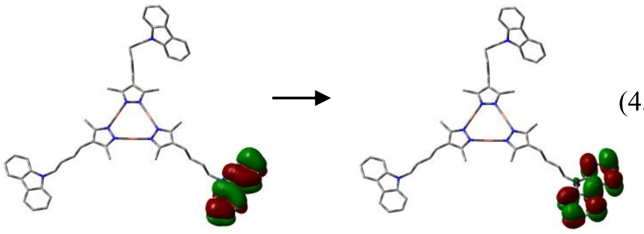 (45%)	^{1}LC
				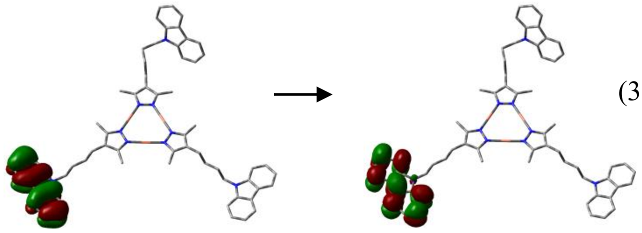 (38%)	
S_8	4.413	268.8	0.1303	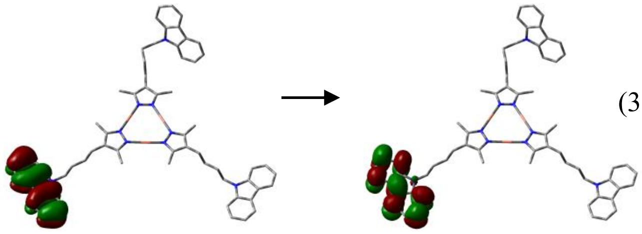 (36%)	^{1}LC

				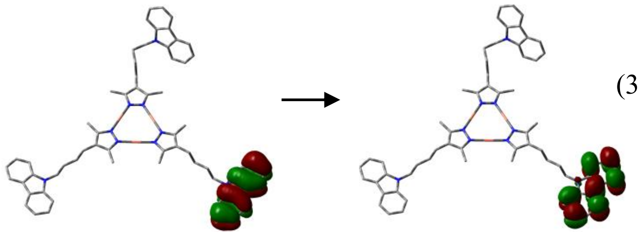 (37%)	
S_{14}	4.858	255.2	0.0420	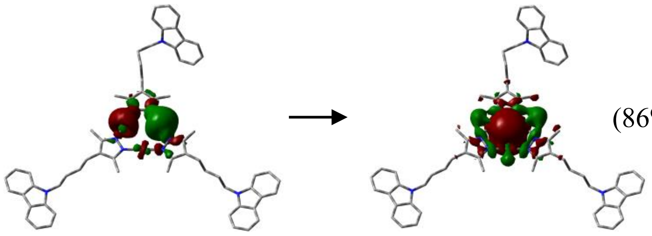 (86%)	^{1}MLCT
S_{16}	4.872	254.5	0.0381	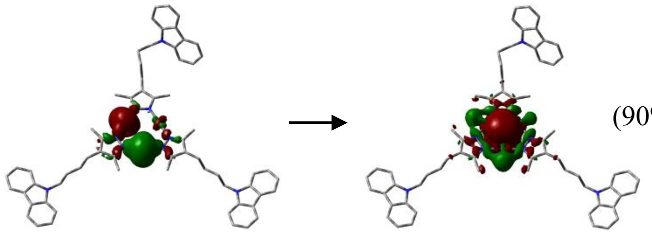 (90%)	^{1}MLCT

Table S5. TDDFT results of the lowest singlet-triplet excited state (ES) for **1a** in T_1 geometry. Major natural transition orbitals (NTO) were demonstrated.

	E /eV	λ /nm	NTO (hole-electron)	Assignment
monomer	2.4533	505.4	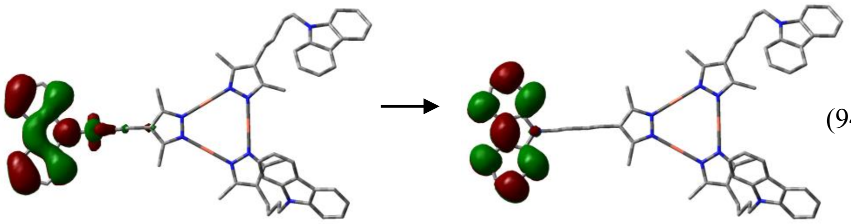 (94%)	^{3}LC
dimer (a)	2.4335	509.6	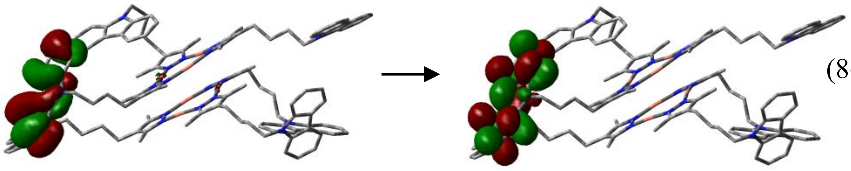 (86%)	^{3}LC
dimer (b)	2.4107	514.4	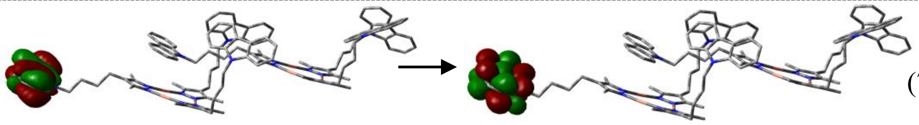 (79%)<br>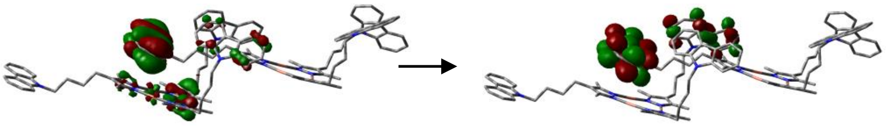 (9%)	^{3}LC<br>^{3}LLCT<br>^{3}MLCT

Table S6. TDDFT results of the lowest singlet-triplet excited state (ES) for **1b** in T_1 geometry. Major natural transition orbitals (NTO) were demonstrated.

	**E /eV**	**λ /nm**	**NTO (hole-electron)**	**Assignment**
monomer	2.4347	509.3	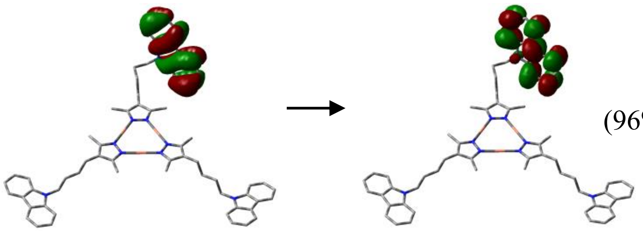 (96%)	^{3}LC
dimer	2.4048	515.6	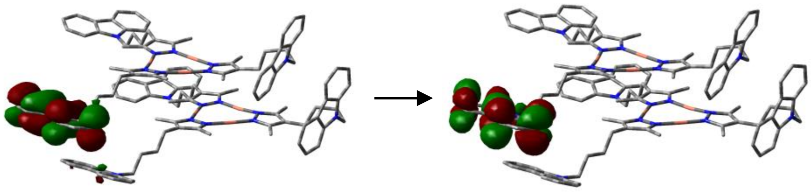 (63%)	^{3}LC
			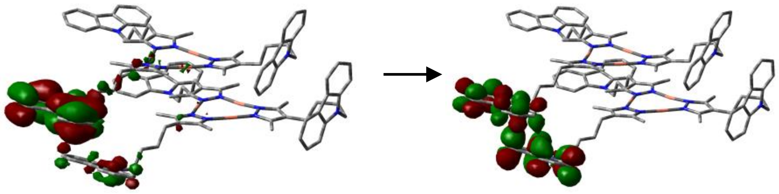 (30%)	

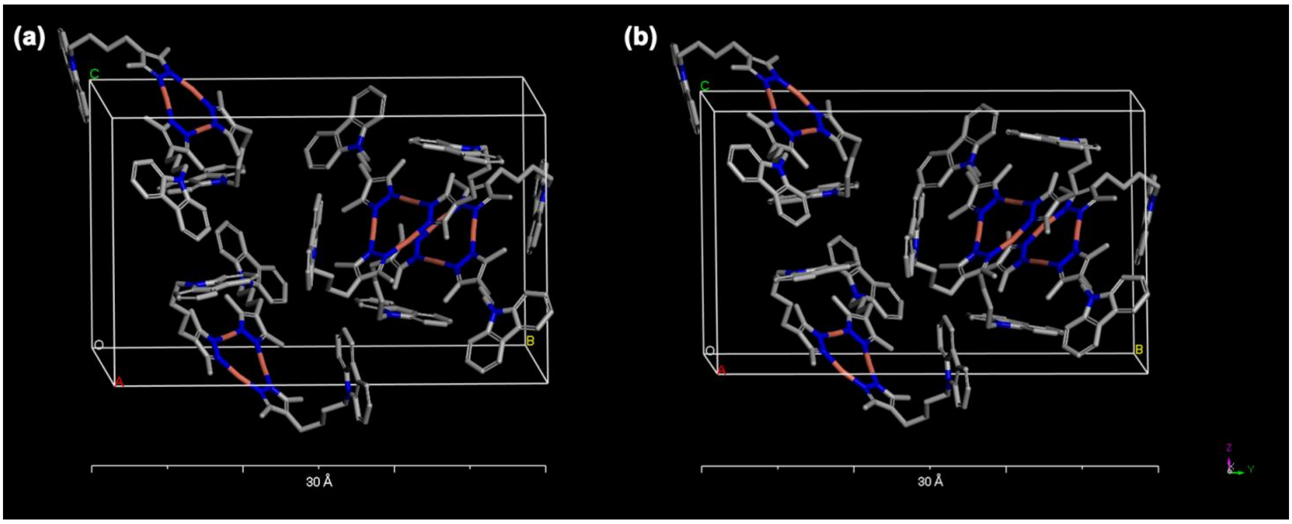

Figure S27. CASTEP optimized structure and unit cell shape under the external pressure of (a) 1 atm and (b) 2.5 GPa for **1a**.

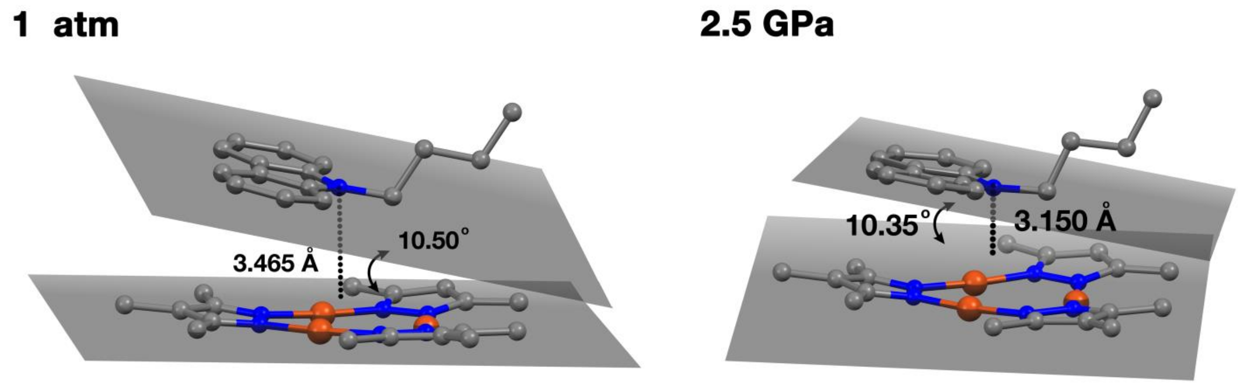

Figure S28. TDDFT calculated T_1 geometry of **1a** at 1 atm and 2.5 GPa. The vertical distances of N atom of **Cz** to **Cu$_3$** plane, and the angles between **Cz** and **Cu$_3$** plane were shown.

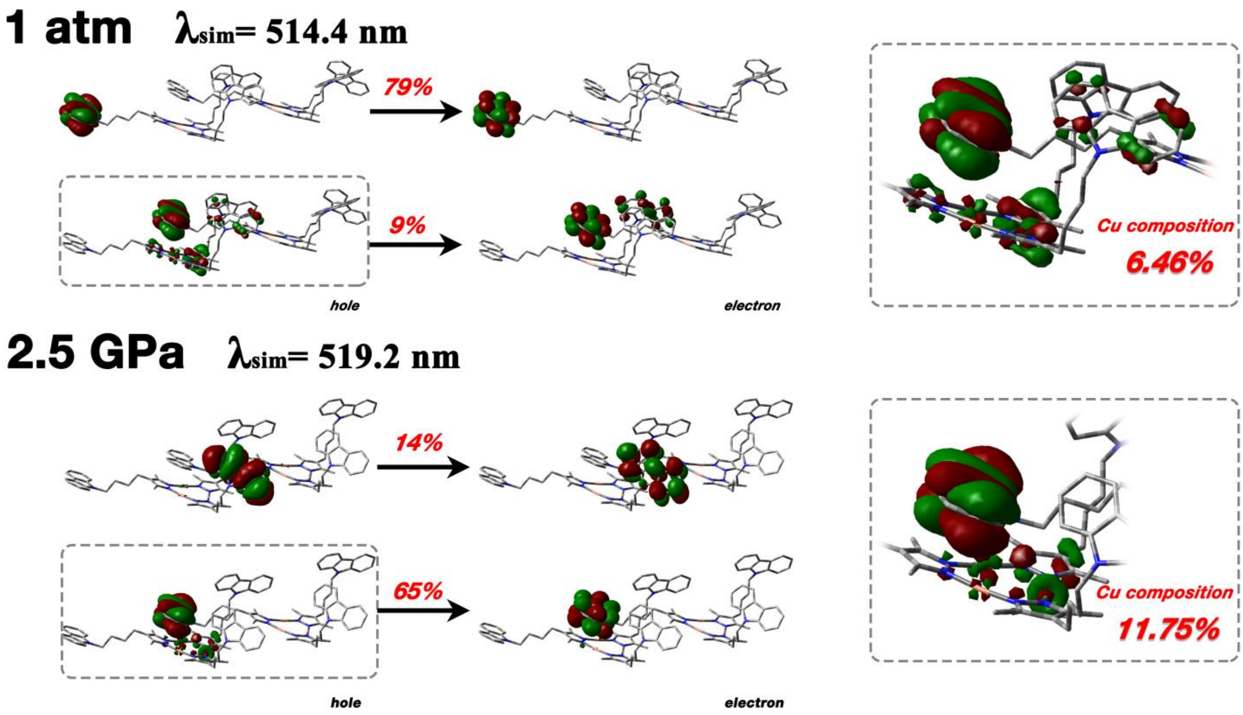

Figure S29. Simulated the lowest triplet (T_1 state) emission energy (λ_{sim}) and corresponding electron transitions of **1a** at 1 atm and 2.5 GPa. Major natural transition orbitals (NTO) and their percentage were demonstrated. The Cu compositions in ^{3}MLCT were also exhibited.

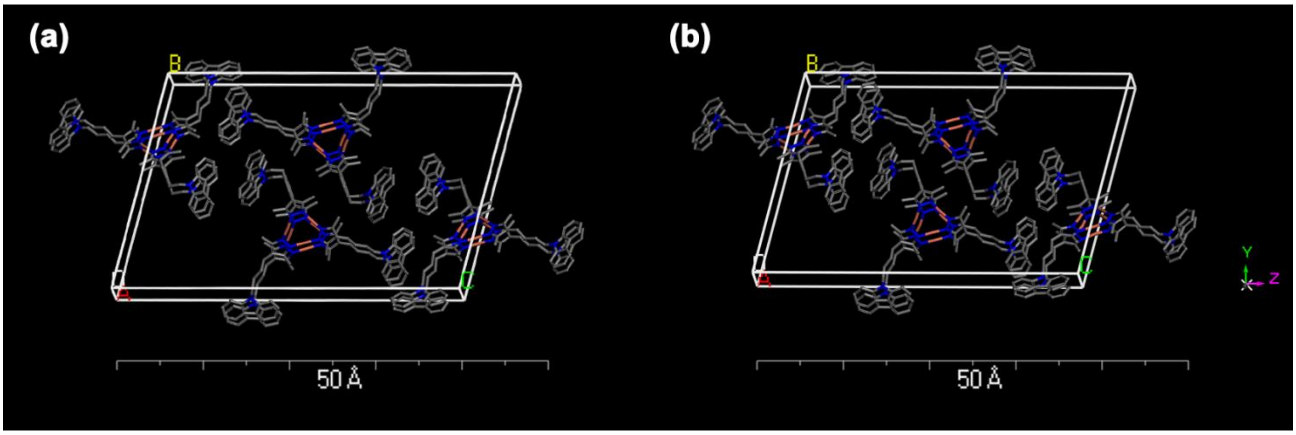

Figure S30. CASTEP optimized structure and unit cell shape under the external pressure of (a) 4GPa and (b) 10GPa for **1b**.

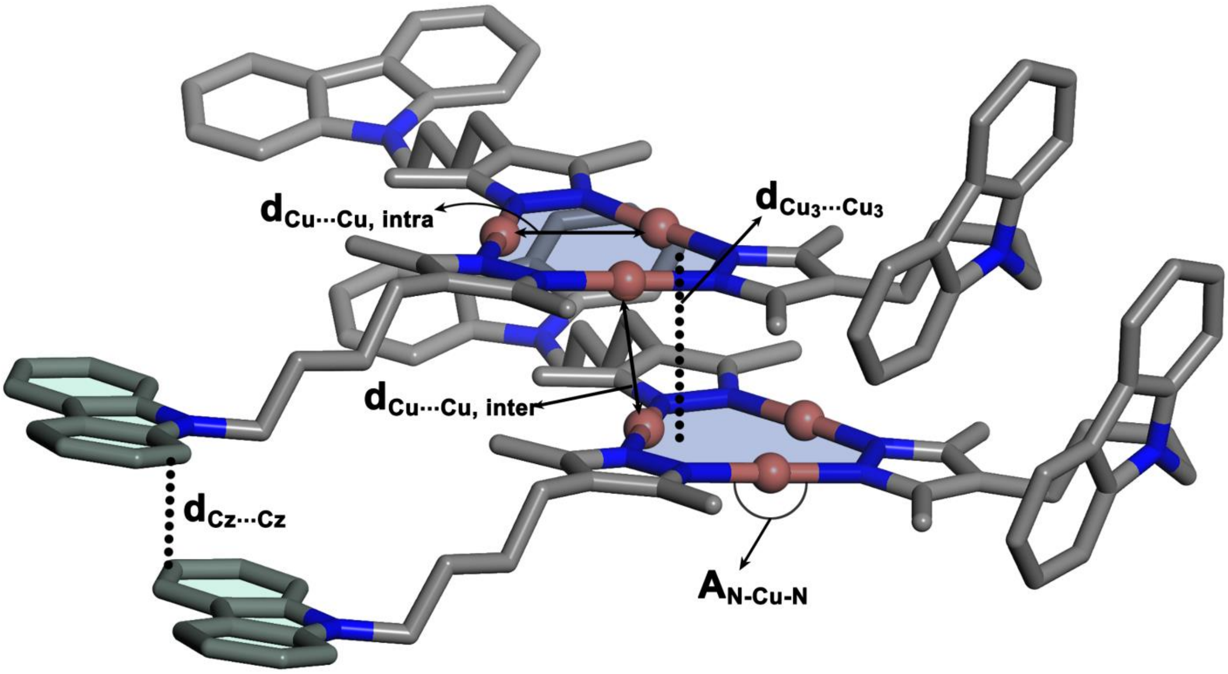

Figure S31. The selected structural parameters of simulated **1b** dimer upon compression.

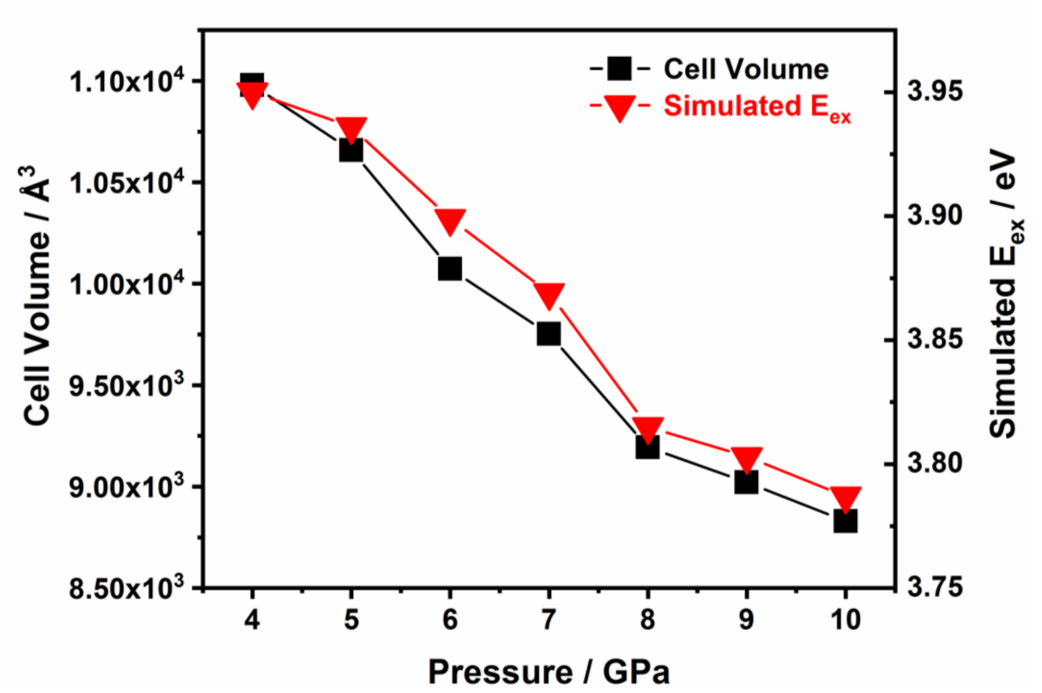

Figure S32. Simulated cell volume and vertical excitation energy against pressure of **1b**.

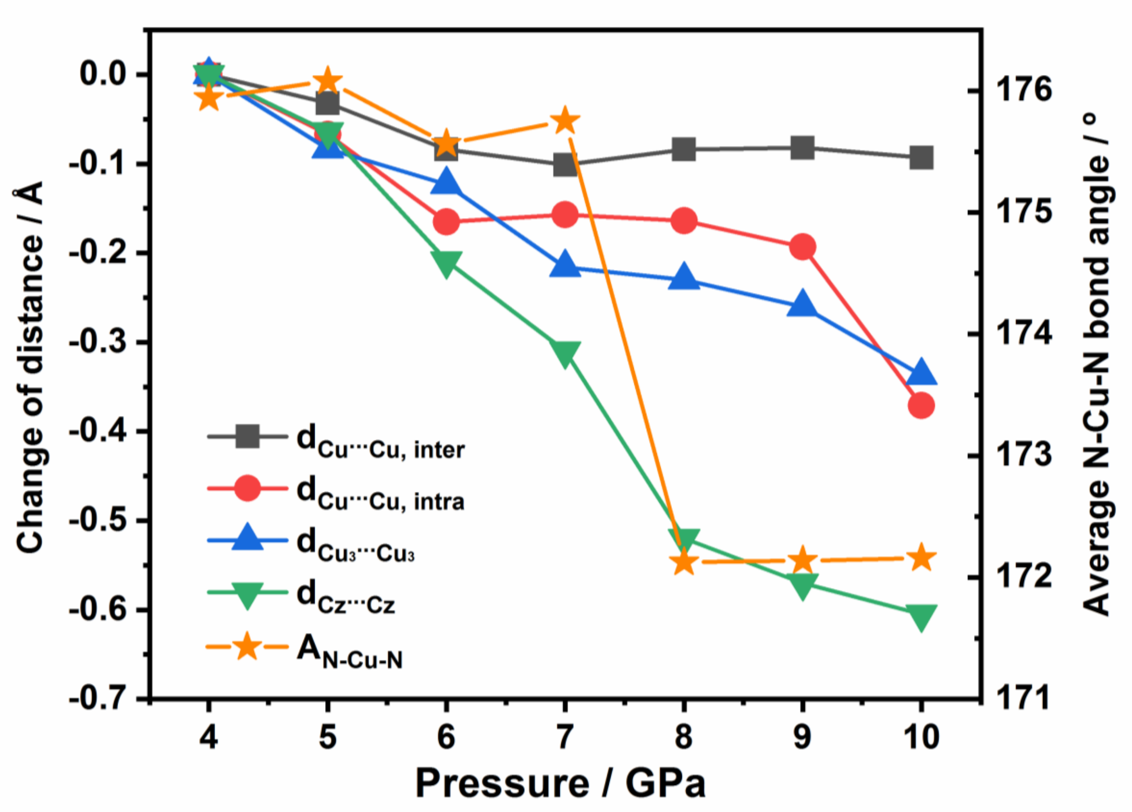

Figure S33. Simulated important structural parameters versus pressure of **1b**.

Table S6. TDDFT results of the lowest lying vertical excitation of **1b** based on the geometry in compression.

	**E / eV**	**λ /nm**	**Transition**	**Assignment**
4 GPa	3.950	313.91	H-2−L	[1]LC
5 GPa	3.936	314.98	H-3−L	[1]LC
6 GPa	3.899	318.02	H-3−L	[1]LC
7 GPa	3.869	320.44	H-3−L	[1]LC
8 GPa	3.815	324.98	H-3−L	[1]LC
9 GPa	3.803	326.02	H-3−L	[1]LC
10 GPa	3.787	327.39	H-3−L	[1]LC

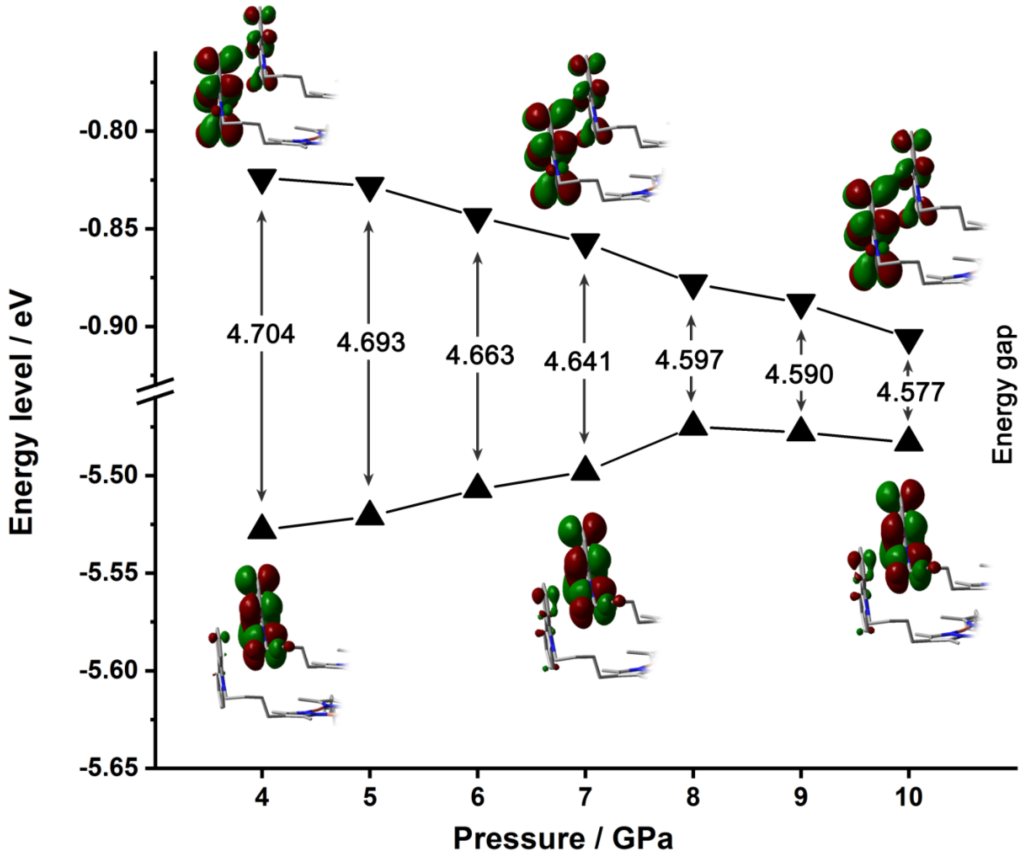

Figure S34. Simulated energy levels of the molecular orbitals (top: LUMO; bottom: HOMO-2/HOMO-3) of **1b** which participating electronic transition under the external pressure of 4 – 10 GPa. Isosurface diagram of MOs at 4, 7 and 10 GPa and energy gap were also displayed.

Reference

(1) Hübschle, C. B.; Sheldrick, G. M.; Dittrich, B. *J. Appl. Crystallogr.* **2011**, *44*, 1281.

(2) Sheldrick, G. M. *Acta Crystallogr. Sect. A: Found. Crystallogr.* **2008**, *64*, 112.

(3) Perdew, J. P.; Burke, K.; Ernzerhof, M. *Phys. Rev. Lett.* **1996**, *77*, 3865.

(4) Clark, S. J.; Segall, M. D.; Pickard, C. J.; Hasnip, P. J.; Probert, M. I. J.; Refson, K.; Payne, M. C. *Zeitschrift für Kristallographie - Crystalline Materials* **2005**, *220*, 567.

(5) M. J. Frisch, G. W. T., H. B. Schlegel, G. E. Scuseria, M. A. Robb, J. R. Cheeseman, G. Scalmani, V. Barone, B. Mennucci, G. A. Petersson, H. Nakatsuji, M. Caricato, X. Li, H. P. Hratchian, A. F. Izmaylov, J. Bloino, G. Zheng, J. L. Sonnenberg, M. Hada, M. Ehara, K. Toyota, R. Fukuda, J. Hasegawa, M. Ishida, T. Nakajima, Y. Honda, O. Kitao, H. Nakai, T. Vreven, J. A. Montgomery, Jr., J. E. Peralta, F. Ogliaro, M. Bearpark, J. J. Heyd, E. Brothers, K. N. Kudin, V. N. Staroverov, R. Kobayashi, J. Normand, K. Raghavachari, A. Rendell, J. C. Burant, S. S. Iyengar, J. Tomasi, M. Cossi, N. Rega, J. M. Millam, M. Klene, J. E. Knox, J. B. Cross, V. Bakken, C. Adamo, J. Jaramillo, R. Gomperts, R. E. Stratmann, O. Yazyev, A. J. Austin, R. Cammi, C. Pomelli, J. W. Ochterski, R. L. Martin, K. Morokuma, V. G. Zakrzewski, G. A. Voth, P. Salvador, J. J. Dannenberg, S. Dapprich, A. D. Daniels, Ö. Farkas, J. B. Foresman, J. V. Ortiz, J. Cioslowski, and D. J. Fox, *Gaussian, Inc.Wallingford CT,* **2009**.

(6) Roy, L. E.; Hay, P. J.; Martin, R. L. *J. Chem. Theory. Comput.* **2008**, *4*, 1029.

(7) Hariharan, P. C.; Pople, J. A. *Theor. Chim. Acta* **1973**, *28*, 213.

(8) Adamo, C.; Barone, V. *J. Chem. Phy.* **1999**, *110*, 6158.

(9) Lu, T.; Chen, F. *J. Comput. Chem.* **2012**, *33*, 580.
